# Design, synthesis, molecular modelling and biological evaluation of novel 3-(2-naphthyl)-1-phenyl-1H-pyrazole derivatives as potent antioxidants and 15-Lipoxygenase inhibitors

**DOI:** 10.1080/14756366.2020.1742116

**Published:** 2020-03-27

**Authors:** Sahar A. Ali, Samir Mohamed Awad, Ahmed Mohammed Said, Shahenda Mahgoub, Heba Taha, Naglaa Mohamed Ahmed

**Affiliations:** a Department of Biochemistry and Molecular Biology, Faculty of Pharmacy, Helwan University, Ein-Helwan, Cairo, Egypt; b Department of Pharmaceutical Organic Chemistry, Faculty of Pharmacy, Helwan University, Ein-Helwan, Cairo, Egypt; c Department of Pharmacy, Al-Zahrawi University College, Karbala, Iraq; d Department of Chemistry, University at Buffalo, The State University of New York, Buffalo, NY, USA

**Keywords:** Pyrazole, hybrids, antioxidant activity, scavenging activity, 15-lipoxygenase inhibitors

## Abstract

Oxidative stress is one of the main causes of significant severe diseases. The discovery of new potent antioxidants with high efficiency and low toxicity is a great demand in the field of medicinal chemistry. Herein, we report the design, synthesis molecular modelling and biological evaluation of novel hybrids containing pyrazole, naphthalene and pyrazoline/isoxazoline moiety. Chalcones **2a–e** were synthesized efficiently and were used as starting materials for synthesis of a variety of heterocycles. A novel series of pyrazoline **3a–e**, phenylpyrazoline **4a–e**, isoxazoline **5a–e** and pyrazoline carbothioamide derivatives **6a–e** were synthesized and screened for *in vitro* antioxidant activity using 2,2-diphenyl-1-picrylhydrazyl (DPPH), nitric oxide (NO) and superoxide radical scavenging assay as well as 15-lipoxygenase (15-LOX) inhibition activity. Compounds **3a, 4e, 5b, 5c, 6a, 6c,** and **6e** showed excellent radical scavenging activity in all three methods in comparison with ascorbic acid and 15-LOX inhibition potency using quercetin as standard then were subjected to *in vivo* study. Catalase (CAT) activity, glutathione (GSH) and malondialdehyde (MDA) levels were assayed in liver of treated rats. Compounds **5b, 5c,** and **6e** showed significant *in vivo* antioxidant potentials compared to control group at dose of 100 mg/kg B.W. Molecular docking of compound **6a** endorsed its proper binding at the active site pocket of the human 15-LOX which explains its potent antioxidant activity in comparison with standard ascorbic acid.

## Graphical Abstract

**Figure:**
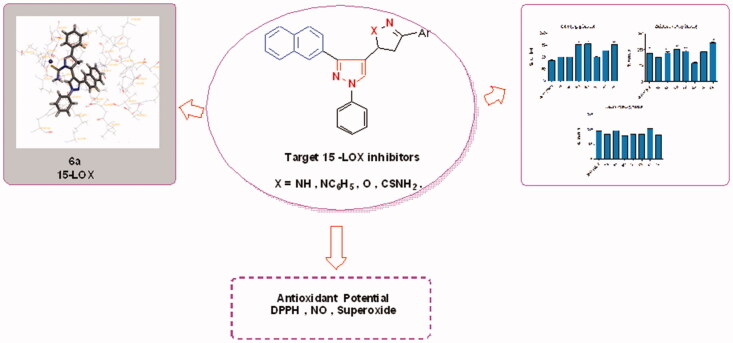

## 1. Introduction

Oxidative stress is one of the main causes of significant severe diseases, i.e. cancer, aging, atherosclerosis, hypertension, inflammation, renal disorders, liver disorders, rheumatoid arthritis, neurological disorders, cardiovascular, autoimmune diseases and neurodegenerative disorders such as Alzheimer’s, Huntington’s diseases and Parkinson’s diseases^1–3^. It is caused by the human body excessive production of reactive oxygen species (ROS) and nitrogen reactive species (NRS) such as hydrogen peroxides (H_2_O_2_) and free radicals. The balance between the production and neutralization of ROS by antioxidants is very delicate^4^. Every day a human cell is targeted by ROS, the hydroxyl radical (**^.^**OH), and other species inducing oxidative stress^5^. Free radicals (atoms, molecules or ions contain an unpaired electron) are highly unstable and very reactive species that are able to create ROS such as **^.^**OH, hydroperoxyl radical (HO_2_**^.^**), superoxide anion (**^.^**O_2_^−^), nitric oxide (NO), singlet oxygen (O) and H_2_O_2_ as well as nitrogen reactive species (RNS) and reactive sulphur species (RSS). These species are generated either internally from normal metabolic activities or external factors, such as smoking, environmental pollutants and radiation, that promote the production of free radicals. The main human body targets of ROS, RNS and RSS are sugars, proteins, lipids, DNA and RNA molecules^6^. High concentrations of such species can cause damage to the normal cell structures, embedded proteins, carbohydrates, lipids, and disrupt nitrogen bases of nucleic acids leading to the above-mentioned diseases. The human body creates a primary defence antioxidant mechanism for the detoxification of the formed free radicals. This mechanism involves three enzymes: superoxide dismutase (SOD), catalase (CAT) and glutathione peroxidase (GPx)^7^. The action of these enzymes is more prominent in the presence of antioxidant agents. Antioxidants are molecules that delay and prevent oxidative damage to a target molecule. In addition, antioxidants inhibit ROS production and diminish oxidative stress^8^. The essential defence role of antioxidants in the human body is via scavenging or regulating the production and elimination of ROS and RNS. The presence of favourable balance between ROS and antioxidants is important for healthy tissues and proper physiological function. It is also well known that the balance between free radicals, antioxidants and co-factors can contribute to the delay of the aging process, reduce the incidence of diseases and thus contributing to a better quality of life. Therefore, the discovery of new potent antioxidants with high efficiency and low toxicity is of a great demand in the field of medicinal chemistry.

**Figure 1.**
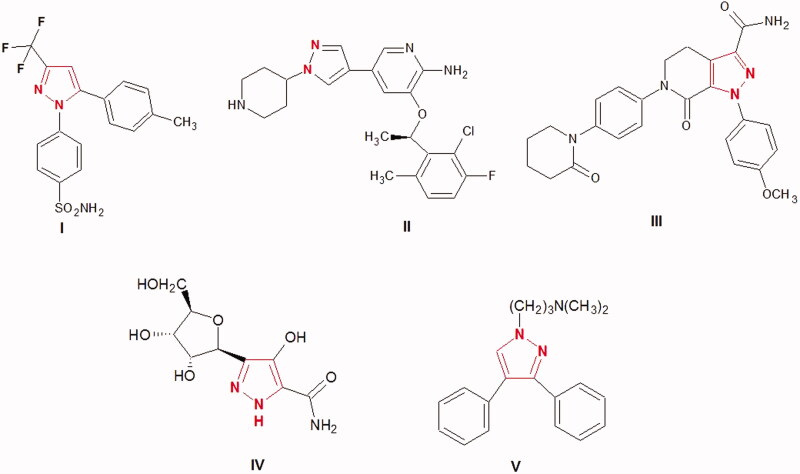
Biologically active compounds have pyrazole ring.

Pyrazole ring is an important scaffold in medicinal chemistry. Pyrazole is a five-membered heterocyclic ring that consists of three carbons and two adjacent nitrogen atoms. Pyrazole derivatives have received considerable attention due to their remarkable broad spectrum of medicinal and pharmacological activities i.e. anticancer^9^, antiviral^10^, anti‐tubercular^11^, anti-microbial^12^, antimalarial^13^, anti-inflammatory^14^, antihypertensive^15^, anti-Alzheimer’s^16^, antipsychotic^17^, and antiparkinsonian^18^. Various drugs that have pyrazole ring are available in the market with diverse medicinal activities i.e. celecoxib **I** as anti-inflammatory^19^, Crizotinib **II** as anticancer^20^, Apixaban **III** as anticoagulant^21^, Pyrazofurin **IV** as anticancer, antibiotic^22^ and Fezolamine **V** as antidepressant^23^ (Figure 1).

**Figure 2.**
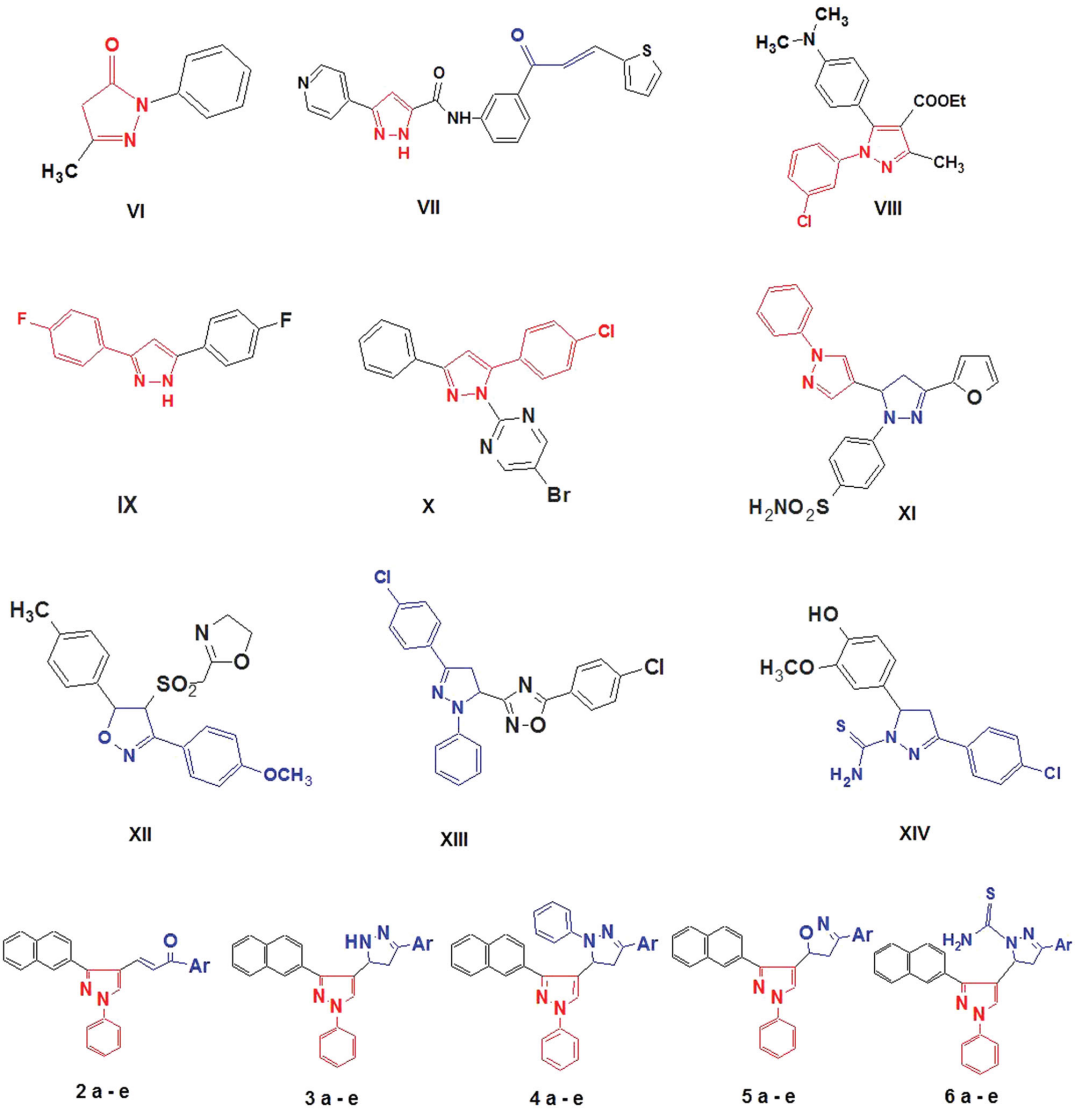
Structure of the lead antioxidant pyrazole derivatives and the designed target compounds **2–6**.

The pyrazole (1,2-diazole) has antioxidant activity and can prevent oxidative stress by increasing antioxidant enzymes, such as GPx, and diminishing the lipid peroxidation process. Examples for the pharmacological effects of 1,2-diazole or its related drugs. 1,2-Diazole was found to be effective in preventing nephrotoxicity caused by the anti-neoplastic drug cisplatin^24^. Edaravone **VI** (Figure 2) is a novel antioxidant that has been used for patients in cerebral infarction as support therapy for stroke^25^^,^^26^ and improves ischemia/reperfusion-induced hepatic energy metabolism^27^.

Recently, the study of pyrazole as a pharmacophore for the development of potential antioxidants has led to the synthesis of several compounds containing pyrazole core in their structures. Among the reported synthetic pyrazoles: 3-(Pyridin-4-yl)-1H-pyrazole-5-carboxamide chalcones **VII** showed potent radical scavenging activity (RSA) against 2,2-diphenyl-1-picrylhydrazyl (DPPH) radical^28^, Moreover, in comparison with the standard ascorbic acid, 1,5-diarylpyrazoles **VIII** showed good DPPH RSA^29^. It was found that 3,5-diarylpyrazole **IX** has shown potent RSA as well. The antioxidant activity of pyrazole is attributed to the presence of NH proton of the pyrazole moiety^30^. In addition, 3,5-diarylpyrazoline derivative **X** showed excellent RSA using DPPH, **^.^**OH, **^.^**O_2_**^−^** and NO anion assays, compared to butylated hydroxy toluene (BHT7)^31^. Derivatives of pyrazole such as bipyrazole **XI** showed good scavenging activity (19%, BHT7=20%) in the DPPH assay at 10^−4 ^M concentration^32^. As well as, Bis-isoxazoline **XII** showed good RSA using DPPH, NO and H_2_O_2_ methods in comparison with ascorbic ascid^33^. Moreover, Pyrazolyl-1,2,4-oxadiazoles **XIII** possessed potent DPPH RSA^34^. 4,5-Dihydropyrazole-1-carbothioamide derivative **XIV** exhibited good antioxidant activity at low concentrations (0.25 mg/mL) in DPPH method^35^ (Figure 2).

**Figure 3.**
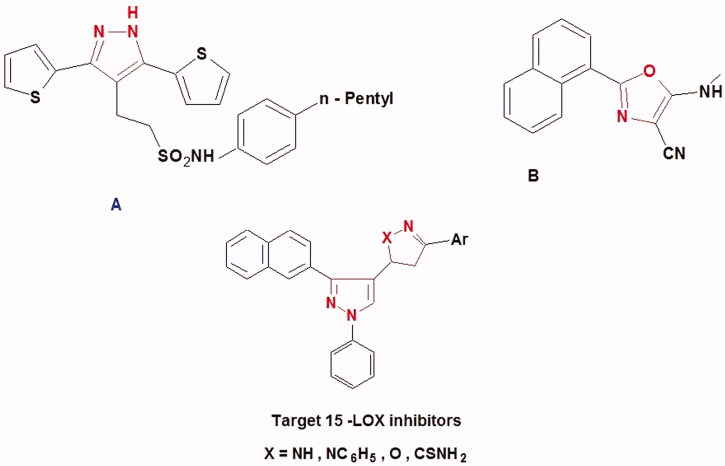
Design strategy of new pyrazole hybrid compounds as 15-LOX inhibitors.

The mechanism of action of antioxidants can be through various pathways such as free radical scavengers (preventive oxidants) and as lipoxygenase inhibitors (pro-oxidative enzymes)^36^^,^^37^. 15-Lipoxygenases (15-LOXes)^38^^,^^39^ are a unique class of non-heme iron containing enzymes that catalyse the peroxidation of polyunsaturated fatty acids such as arachidonic acid (AA) and linoleic acid to their related hydroperoxides. In addition, 15-LOXes are involved in various human diseases. 15-lipoxygenase-1 (15-LOX-1) has been recently documented as a target for reduction of the biosynthesis of eoxines, pro-inflammatory mediator^40^ and cancer promoter^41^. Also, it was reported that 15-LOX participates in the oxidative modification of low-density lipoproteins (LDLs) that leads to the progress of atherosclerosis^42^. Moreover, human 15-LOX-1 is one of the key mediators in neurodegenerative diseases such as Alzheimer’s disease^43^. There has been some literature work targeting 15-LOX-1. It was reported that 3,4,5-trisubstituted pyrazole (**A**) was found to work as a potent rabbit 15-LOX-1 inhibitor^44^. Recently, oxazole derivative (ML351) (**B**) showed novel 15- LOX inhibition with potent activity against human 15-LOX-1 in both a cellular and an *in vivo* model of stroke^45^ (Figure 3).

In this study, we report the design, synthesis and biological evaluation of a hybrid scaffold in which 3-naphthyl pyrazole is substituted with pyrazoline/isoxazoline ring at position 3 to generate novel and new derivatives of 3-(2-naphthyl)-1-phenyl-1*H*-pyrazole (Figure 3). These novel hybrid derivatives were tested against 15-LOX enzymatic assay. Moreover, these compounds were evaluated for their potential as antioxidants in DPPH, NO, and superoxide scavenging assays as well as *in vivo* antioxidant activity using CAT, glutathione (GSH) and lipid peroxidation (MDA) assays. The results of *in vitro* antioxidant activity of the newly designed hybrids and their 15-LOX inhibitory activity would identify the required antioxidant parameters that are most reliable in the design of 15-LOX inhibitors for the future studies. The structure–activity relationship (SAR) and possible mechanisms of action of these derivatives were also investigated.

## 2. Materials and methods

### 2.1. Instruments

Melting points were determined with Electro-thermal IA 9100 apparatus (Shimadzu, Japan) and the values given were uncorrected. Fourier-transform infrared spectroscopy (FT-IR) spectra were recorded as KBr pellets on a Perkin-Elmer 1650 spectrophotometer (USA), Faculty of Science, Cairo University, Cairo, Egypt. Proton nuclear magnetic resonance (^1^HNMR) and carbon-13 nuclear magnetic resonance (^13^C-NMR) spectra were recorded in dimethyl sulfoxide-d6 (DMSO-d6) on a Varian Mercury (300 MHz) spectrometer (Varian UK) using TMS as internal standard and chemical shifts were given as ppm (Faculty of Science, Cairo University, Cairo, Egypt). Mass spectra were carried out using 70 eV EI Ms-QP 1000 EX (Shimadzu, Japan), Faculty of Science, Cairo University, and Cairo, Egypt. Microanalyses were performed on Vario, Elementar apparatus (Shimadzu, Japan), Organic Microanalysis Unit, Faculty of Science, Cairo University, Cairo, Egypt and the results were within the accepted range (0.40) of the calculated values. Column Chromatography was performed on (Merck) Silica gel 60 (particle size 0.06–0.20 mm).

### 2.2. Chemistry

***3-(2-Naphthyl)-1-phenyl-1H-pyrazole-4-carbaldehyde (1).*** The titled compound **1** was synthesized according to the literature procedure^46^^,^^47^. A mixture of β-acetyl naphthalene (0.03 mol) and 0.04 mol of phenyl hydrazine (0.03 mol) in absolute ethanol (50 mL) and few drops of glacial acetic acid were heated on water bath for 30 min. The progress of reaction was monitored by thin-layer chromatography (TLC) using hexane and ethanol (90:10). Cooling the mixture and filtering the formed precipitate that was dried and crystallized from ethanol, a pure phenyl hydrazone was obtained. Pyrazole-4-carbaldehyde was carried out by the application of two moles of cold solution of Vismyeir–Haack (VH) reagent (DMF-POCl_3_) with the phenyl hydrazone (0.01 mol) in DMF (3 mL). The reaction mixture was stirred at 70–80 °C for 5–6 h. The progress of reaction was monitored by TLC using hexane and ethanol (90:10). The reaction was cooled to room temperature, then poured into cold water and a saturated solution of sodium bicarbonate was added to neutralise the mixture. The white solid obtained was filtered followed by washing with water.

***3-(3-Naphthalen-2-yl-1-phenyl-1H-pyrazole-4-yl)-1-aryl propenone (2a–e).*** A mixture of 4-substituted acetophenone (0.03 mol) and the aldehyde **1** (0.03 mol) in 25 mL 50% alcoholic NaOH solution were stirred at room temperature for 24 h, then the solution was cooled, poured on ice/water acidified with dil. HCl. The produced solid was filtered off, dried and crystallized from ethanol to give compounds **2a–e**.

***3-(3-Naphthalen-2-yl-1-phenyl-1H-pyrazole-4-yl)-1-phenyl propenone (2a).***Yellow solid, yield 81%, m.p.158–159 °C. IR (KBr) v_max_ (cm^−1^): 3150 (CH–Ar), 1695 (C=O), 1604 (C=N). ^1^H NMR (300 MHz, DMSO-d_6_) *δ*: 6.5 (d, 1H, *J*=6.8 Hz, –CH=CH–), 6.6 (d, 1H, *J*=19.1 Hz, –CH=CH–), 6.8 (s, 1H, pyrazole), 7.1–7.9 (m, 17H, Ar–H).^13^C NMR (300 MHz, DMSO-d_6_) *δ*: 105.0 (pyrazole-C4), 126.0 (pyrazole-C5), 114.7–140.1 (aromatic Cs), 129. 26, 142.8 (CH=CH), 160.0 (pyrazole-C3), 187.0 (C=O). MS (EI‏): m/z: 400 [M^+^] (20%). Anal. Calcd for C_28_H_20_N_2_O (400.471): C, 83.98; H, 5.03; N, 7.00; Found: C,83. 77; H, 5.15; N, 6.93.

***1-(4-Methoxyphenyl)-3-(3-naphthalen-2-yl-1-phenyl-1H-pyrazole-4-yl)-propenone (2b).*** Brown solid, yield 85%, m.p.187–188 °C. IR (KBr) v_max_ (cm^−1^): 2970 (CH-sp^3^), 3157 (CH–Ar), 1691 (C=O), 1605 (C=N). ^1^H NMR (300 MHz, DMSO-d_6_) *δ*: 3.3 (s, 3H, OCH_3_), 7.0 (d, 1H, *J*=6.5 Hz, –CH=CH–), 7.4 (d, 1H, *J*=18.1 Hz, –CH=CH–), 6.6 (s, 1H, pyrazole), 7.5-8.55 (m, 16H, Ar–H). ^13^C NMR (300 MHz, DMSO-d_6_) *δ*: 55.87 (OCH_3_), 105.21 (pyrazole-C4), 126.66 (pyrazole-C5), 113.33–145.0 (aromatic carbons), 129.30, 148.40 (CH=CH), 161.0 (pyrazole-C3), 183.0 (C=O). MS (EI‏): m/z: 430 [M^+^] (20%). Anal. Calcd for C_29_H_22_N_2_O_2_ (430.497): C,80.91; H, 5.15; N, 6.51. Found: C,80.78; H,5.17; N,6.72.

***3-(3-Naphthalen-2-yl-1-phenyl-1H-pyrazol-4-yl)-1-p-tolyl propenone (2c).*** Yellow solid, yield 80%, m.p.146–147 °C. IR (KBr) v_max_ (cm^−1^): 2975 (CH-sp^3^), 3160 (CH–Ar), 1696 (C=O), 1605 (C=N). ^1^H NMR (300 MHz, DMSO-d_6_) *δ*: 2.3 (s, 3H, CH_3_), 6.5 (d, 1H, *J*=6.7 Hz, –CH=CH–), 6.7 (d, 1H, *J*=18.1 Hz, –CH=CH–), 6.8 (s, 1H, pyrazole), 7.0–7.9 (m, 16H, Ar–H). ^13^C NMR (300 MHz, DMSO-d_6_) *δ*: 20.7 (CH_3_), 105.0 (pyrazole-C4), 126.50 (pyrazole-C5), 112.7–142.1 (aromatic carbons), 126.26, 140.8 (CH=CH), 163.0 (pyrazole-C3), 187.0 (C=O). MS (EI‏): m/z: 414 [M^+^] (17.7%). Anal. Calcd for C_29_H_22_ N_2_O (414.49): C,84.03; H, 5.35; N, 6.76; Found: C,84.19; H,5.27; N,6.67.

***1-(4-chlorophenyl)-3-(3-naphthalen-2-yl-1-phenyl-1H-pyrazol-4-yl)propenone (2d).*** Yellow solid, yield 77%, m.p.161–162 °C. IR (KBr) v_max_ (cm^−1^): 3157 (CH–Ar), 1692 (C=O), 1655 (C=N). ^1^H NMR (300 MHz, DMSO-d_6_) *δ*: 6.4 (d, 1H, *J*=6.6 Hz, –CH=CH–), 6.8 (d, 1H, *J*=17.1 Hz, –CH=CH–), 6.9 (s, 1H, pyrazole), 7.1–7.8 (m, 16H, Ar–H). ^13^C NMR (300 MHz, DMSO-d_6_) *δ*:105.5 (pyrazole-C4), 126.2 (pyrazole-C5), 115.7–145.1 (aromatic carbons), 126.2,141.1 (CH=CH), 160.3 (pyrazole-C3), 187.0 (C=O). MS (EI‏): m/z: 434 [M^+^] (20.1%), 436 (M + 2, 6.7%). Anal. Calcd for C_28_H_19_ClN_2_O: (434.916): C, 77.33; H, 4.40; N, 6.44; Found: C,77.29; H, 4.45; N,6.47.

***1-(3,4-Dichlorophenyl)-3-(3-naphthalen-2-yl-1-phenyl-1H-pyrazol-4-yl)propenone (2e).*** Yellow solid, yield 79%, m.p.168-169 °C. IR (KBr) v_max_ (cm^−1^): 3156 (CH–Ar), 1691 (C=O), 1603 (C=N). ^1^H NMR (300 MHz, DMSO-d_6_) *δ*: 6.2 (d, 1H, *J*=6.6 Hz, –CH=CH–), 6.4 (d, 1H, *J*=18.1 Hz, –CH=CH–), 6.7 (s, 1H, pyrazole), 7.0–7.9 (m,15H,Ar–H). ^13 ^C NMR (300 MHz, DMSO-d_6_) *δ*: 105.0 (pyrazole-C4), 126.0 (pyrazole-C5), 113.3–140.4 (aromatic carbons), 126.2,142.2 (CH=CH), 160.2 (pyrazole-C3), 187.0 (C=O). MS(EI‏): m/z: 469 [M^+^] (15.3%), 471 (M + 2, 5.1%). Anal. Calcd for C_28_H_18_Cl_2_N_2_O (469.36): C,71.65; H, 3.87; N, 5.97; Found: C,71.55; H, 3.85; N,5.83.

***3-Naphthalen-2-yl-5-aryl,1’-phenyl-3,4-dihydro-2H,1H’-[3,4] bipyrazole (3a–e).*** A solution of **(2a–e)** (1.0 mmol) and hydrazine hydrate 99% (1.0 mmol) in absolute ethanol (15 mL) was refluxed for 6-8 h. The resulting solution was concentrated, cooled, the solid obtained was filtered off and recrystallized from ethanol to give compounds **3a–e**.

***3′-Naphthalen-2-yl-5,1′-diphenyl-3,4-dihydro-2H,1’H-[3,4′]bipyrazole (3a).*** Yellow solid, yield 61%, m.p.172–173 °C. IR (KBr) v_max_ (cm^−1^): 2960 (CH-sp^3^), 3052 (CH–Ar), 3439 (NH), 1593 (C=N). ^1^H NMR (300 MHz, DMSO-d_6_) *δ*: 3.10–3.88 (dd, 2H, pyrazoline -C4-H), 5.26 (t, *J =* 11.5 Hz, 1H, pyrazoline-C5-H), 6.8 (s,1H,pyrazole),7.0–8.0 (m, 17H, Ar–H), 8.3 (s, 1H, NH-pyrazoline, D_2_O exchangeable). ^13^C NMR (300 MHz, DMSO-d_6_) *δ*: 39.79 (pyrazoline-C4), 55.87 (pyrazoline-C5), 105.0 (pyrazole-C4), 125.99 (pyrazole-C5), 118.68–145.05 (aromatic carbons), 150.71 (pyrazoline-C3), 158.01 (pyrazole-C3). MS (EI‏): m/z:414 [M^+^] (13.7%). Anal. Calcd for C_28_H_22_N_4_ (414.50): C,81.13; H, 5.35; N, 13.52; Found: C,81.17; H,5.23; N,13.43.

***5-(4-Methoxyphenyl)-3′-naphthalen-2-yl-1′-phenyl-3,4-dihydro-2H,1’H-[3,4′] bipyrazole (3b).*** Brown solid, yield 67%, m.p.177–178 °C. IR (KBr) v_max_ (cm^−1^): 2965 (CH-sp^3^), 3163 (CH–Ar), 3356 (NH), 1605 (C=N). ^1^H NMR (300 MHz, DMSO-d_6_) *δ*: 3.22–3.40 (dd, 2H, pyrazoline-C4-H), 3.9 (s, 3H, OCH_3_), 5.20 (t, *J =* 11.5 Hz, 1H, pyrazoline-C5-H), 6.7(s, 1H, pyrazole), 6.9–7.9 (m, 16H, Ar–H), 8.0 (s, 1H, NH-pyrazoline, D_2_O exchangeable). ^13^C NMR (300 MHz, DMSO-d_6_) *δ*: 38.30 (pyrazoline-C4), 55.60 (pyrazoline-C5), 56.0(OCH_3_), 105.0 (pyrazole-C4), 126.0 (pyrazole-C5), 112.2–141.1 (aromatic carbons), 148.54 (pyrazoline-C3), 159.0 (pyrazole-C3). MS (EI‏): m/z: 444 [M^+^] (14.3%). Anal. Calcd for C_29_H_24_N_4_O: (444.53):C,78.36; H, 5.44; N, 12.60; Found: C,78. 33; H,5.43; N,12.70.

***3’-Naphthalen-2-yl-1’-phenyl-5-p-tolyl-3,4-dihydro-2H, 1’H -[3,4’]bipyrazole (3c).*** Yellow crystals, yield 66%, m.p.187-188 °C. IR (KBr) v_max_ (cm^−1^): 2963 (CH-sp^3^), 3164 (CH–Ar), 3357 (NH), 1605 (C=N). ^1^H NMR (300 MHz, DMSO-d6) *δ*: 2.5 (s, 3H, CH_3_), 3.20–3.51 (dd, 2H, pyrazoline-C4-H), 5.3 (t, *J =* 11.5 Hz, 1H, pyrazoline-C5-H), 6.6 (s, 1H, pyrazole), 7.0-7.9 (m, 16H, Ar–H), 8.0 (s, 1H, NH-pyrazoline, D_2_O exchangeable). ^13^C NMR (300 MHz, DMSO-d_6_) *δ*: 20.7 (CH_3_), 36.30 (pyrazoline-C4), 57.50 (pyrazoline-C5), 105.0 (pyrazole-C4), 125.0 (pyrazole-C5), 115.5-145.1 (aromatic carbons), 148.54 (pyrazoline-C3) 160.0 (pyrazole-C3). MS (EI‏): m/z: 428 [M^+^] (9.8%). Anal. Calcd for C_29_H_24_N_4_ (428.53): C,81. 28; H, 5.65; N, 13.07; Found: C,81.27; H,5.58; N,13.03.

***5-(4-Chlorophenyl)-3′-naphthalen-2-yl-1′-phenyl-3,4-dihydro-2H,1’H-[3,4′] bipyrazole (3d).*** Yellow crystals, yield 75%, m.p.190–191 °C. IR (KBr) v_max_ (cm^−1^): 2979 (CH-sp^3^), 3173 (CH–Ar), 3354 (NH), 1605 (C=N). ^1^H NMR (300 MHz, DMSO-d_6_) *δ*: 3.2-3.5 (dd, 1H, pyrazoline-C4-H), 5.20 (t, *J =* 11.6 Hz, 1H, pyrazoline-C5-H), 6.8 (s,1H,pyrazole), 7-7.8 (m, 16H, Ar–H), 8.01 (s, 1H,NH-pyrazoline, D_2_O exchangeable). ^13^C NMR (300 MHz, DMSO-d_6_) *δ*: 34.30 (pyrazoline-C4), 56.57 (pyrazoline-C5), 105.0 (pyrazole-C4), 127.0 (pyrazole-C5), 117.7–147.1 (aromatic carbons), 148.50 (pyrazoline-C3), 161.0 (pyrazole-C3). MS (EI‏): m/z: 448 [M^+^] (11.8%),450(M + 2,4.1%). Anal. Calcd for C_28_H_21_ClN_4_ (448.95):C,74.91; H,4.71; N,12.48; Found: C,74.87; H, 4.80; N,12. 53.

***5-(3,4-Dichlorophenyl)-3′-naphthalen-2-yl-1′-phenyl-3,4-dihydro-2H,1’H-[3,4′]-bipyrazole (3e).*** Yellow solid, yield 77%, m.p.195–196 °C. IR (KBr) v_max_ (cm^−1^): 2973 (CH-sp^3^), 3175 (CH–Ar), 3351 (NH), 1608 (C=N). ^1^H NMR (300 MHz, DMSO-d_6_) *δ*: 3.4 (dd, *J*=16.5, 11.1 Hz, 1H, pyrazoline-C4-H), 3.2 (dd, *J*=16.4, 11.2 Hz, 1H, pyrazoline-C4-H), 5.23 (t, *J*=11.6 Hz, 1H, pyrazoline-C5-H), 6.8 (s, 1H, pyrazole), 7.2–7.8 (m, 15H, Ar–H), 8.0 (s, 1H, NH-pyrazoline, D_2_Oexchangeable). ^13^C NMR (300 MHz, DMSO-d_6_) *δ*: 38.30 (pyrazoline-C4), 58.50 (pyrazoline-C5), 108.0 (pyrazole-C4), 128.0 (pyrazole-C5), 118.7–140.1 (aromatic carbons), 148.54 (pyrazoline-C3), 158.5 (pyrazole-C3). MS (EI‏): m/z: 482 [M^+^] (14.5%), 484 (M + 2, 4.8%). Anal. Calcd for C_28_H_20_Cl_2_N_4_ (483.39): C,69.57; H, 4.17; N, 11.59; found: C,69.77; H,4.10; N,11.60.

***3-Naphthalen-2-yl-5-aryl,2,1’-diphenyl-3,4-dihydro-2H,1H’-[3,4] bipyrazole (4a–e).*** A solution of (**2a–e**) (1.0 mmol) and phenyl hydrazine (1.0 mmol) in 25 mL ethanol containing 0.5 mL piperidine was refluxed for 6–8 h. The mixture was cooled, filtered off and recrystallized from ethanol to give compounds **4a–e.**

***3′-Naphthalen-2-yl-2,5,1′-triphenyl-3,4-dihydro-2H,1’H-[3,4′]bipyrazole (4a).*** Yellow solid, yield 68%, m.p.187–188 °C. IR (KBr) v_max_ (cm^−1^): 2963 (CH-sp^3^), 3186 (CH–Ar), 1607 (C=N). ^1^H NMR (300 MHz, DMSO-d_6_) *δ*: 3.0–3.4 (dd, 2H, pyrazoline-C4-H), 5.21 (t, *J*=11.5 Hz, 1H, pyrazoline-C5-H), 6.8 (s, 1H, pyrazole), 6.9-7.7 (m, 22H, Ar–H). ^13^C NMR (300 MHz, DMSO-d_6_) *δ*: 35.2 (pyrazoline-C4), 57.50 (pyrazoline-C5), 106.2 (pyrazole-C4), 129.0 (pyrazole-C5), 116.0-140.0 (aromatic carbons), 148.8 (pyrazoline-C3), 158.8 (pyrazole-C3). MS (EI‏): m/z: 490 [M^+^] (9.7%). Anal.Calcd for C_34_H_26_N_4_ (490.6): C, 83.24; H, 5.34; N, 11.42; Found: C,83.47; H,5.33; N,11.49.

***5-(4-Methoxyphenyl)-3′-naphthalen-2-yl-2,1′-diphenyl-3,4-dihydro-2H,1’H-[3,4′] bipyrazole (4b).*** Brown solid, yield 60%, m.p.156–157 °C. IR (KBr) v_max_ (cm^−1^): 2967 (CH-sp^3^), 3187 (CH–Ar), 1600 (C=N). ^1^H NMR (300 MHz, DMSO-d_6_) *δ*: 3.1–3.3 (dd, 2H, pyrazoline-C4-H), 3.8 (s, 3H, OCH_3_), 5.3 (t, *J*=11.5 Hz, 1H, pyrazoline-C5-H), 6.6 (s, 1H, pyrazole), 6.8-7.6 (m, 21H, Ar–H). ^13^C NMR (300 MHz, DMSO-d_6_) *δ*: 35.8 (pyrazoline-C4), 57.6 (pyrazoline-C5), 56.0 (OCH_3_), 106.8 (pyrazole-C4), 129.2 (pyrazole-C5), 116.0 − 140.0 (aromatic carbons), 149.0 (pyrazoline-C3), 159.0 (pyrazole-C3). MS (EI‏): m/z: 520 [M^+^] (11.3%). Anal. Calcd for C_35_H_28_N_4_O (520.62): C,80.74; H, 5.42; N, 10.76. Found: C,80.73; H,5.43; N,10.70.

***3′-Naphthalen-2-yl-2,1′-diphenyl-5-p-tolyl-3,4-dihydro-2H, 1’H -[3,4′]bipyrazole (4c).*** Yellow solid, yield 77%, m.p.198–199 °C. IR (KBr) v_max_ (cm^−1^): 2923 (CH-sp^3^), 3052 (CH–Ar), 1595 (C=N). ^1^H NMR (300 MHz, DMSO-d_6_) *δ*: 2.5 (s, 3H, CH_3_), 3.3–3.6 (dd, 2H, pyrazoline-C4-H), 5.28 (t, *J*=11.5 Hz, 1H, pyrazoline-C5-H), 6.6 (s, 1H, pyrazole), 6.9–8.2 (m, 21H, Ar–H). ^13^C NMR (300 MHz, DMSO-d_6_) *δ*: 20.3 (CH_3_), 39.5 (pyrazoline-C4), 55.8 (pyrazoline-C5), 107.1 (pyrazole-C4), 129.5 (pyrazole-C5), 118.6-140.6 (aromatic carbons), 150.0 (pyrazoline-C3), 159.0 (pyrazole-C3). MS(EI‏): m/z: 504 [M^+^] (13.5%). Anal. Calcd for C_35_H_28_N_4_(504.62): C,83.30; H, 5.59; N, 11.10; Found: C,83.37; H, 6.00; N,11.01.

***5-(4-Chlorophenyl)-3′-naphthalen-2-yl-2,1′-diphenyl-3,4-dihydro-2H,1'H-[3,4′] bipyrazole (4d).*** Yellow solid, yield 71%, m.p.192-193 °C. IR (KBr) v_max_ (cm^−1^): 2978 (CH-sp^3^), 3174 (CH–Ar), 1605 (C=N). ^1^H NMR (300 MHz, DMSO-d_6_) *δ*: 3.3–3.5 (dd, 1H, pyrazoline-C4-H), 5.27 (t, *J*=11.6 Hz, 1H, pyrazoline-C5-H), 6.7 (s, 1H, pyrazole), 7-7.8 (m, 21H, Ar–H). ^13^C NMR (300 MHz, DMSO-d_6_) *δ*:35.9 (pyrazoline-C4), 57.8 (pyrazoline-C5), 106.9 (pyrazole-C4), 129.4 (pyrazole-C5), 116.3–141.1 (aromatic carbons), 149.6 (pyrazoline-C3), 159.6 (pyrazole-C3). MS (EI‏): m/z: 524 [M^+^] (10.8%), 426 (M + 2, 3.5%). Anal. Calcd for C_34_H_25_ClN_4_(525.04): C,77.78; H, 4.80; N, 10.67; Found: C,77.87; H, 4.82; N,10.53.

***5-(3,4-Dichlorophenyl)-3′-naphthalen-2-yl-2,1′-diphenyl-3,4-dihydro-2H,1’H-[3,4′]-bipyrazole (4e).*** Brown solid, yield 75%, m.p.172–173 °C. IR (KBr) v_max_ (cm^−1^): 2974 (CH-sp3), 3176 (CH–Ar), 1605 (C=N). ^1^H NMR (300 MHz, DMSO-d_6_) *δ*: 3.5 (dd, *J*=16.8, 11.1 Hz, 1H, pyrazoline-C4-H), 3.3 (dd, *J*=16.6, 11.2 Hz, 1H, pyrazoline-C4-H), 5.26 (t, *J*=11.6 Hz, 1H, pyrazoline-C5-H), 6.8 (s, 1H, pyrazole), 7–7.8 (m, 20H, Ar–H). ^13^C NMR (300 MHz, DMSO-d_6_) *δ*: 38.80 (pyrazoline-C4), 58.80 (pyrazoline-C5), 108.8 (pyrazole-C4), 128.2(pyrazole-C5), 118.8–140.4 (aromatic carbons), 148.58 (pyrazoline-C3), 158.8 (pyrazole-C3). MS(EI‏): m/z: 558 [M^+^] (16.5%), 560 (M + 2, 5.3%). Anal. Calcd for C_34_H_24_Cl_2_N_4_ (559.48): C,72.99; H, 4.32; N, 10.01; Found: C,72.96; H,4.30; N,10.10.

***5-(3-Naphthalen-2-yl-1-phenyl-1H-pyrazol-4-yl)-3-aryl-4,5-dihydro-isoxazole (5a–e).*** A solution (**2a–e**) (1.0 mmol), and hydroxylamine HCl 99% (1.0 mmol) in absolute ethanol (15 mL) with 0.5 mL piperidine was refluxed for 8–10 h. The resulting solution was concentrated, cooled, the solid obtained was filtered off and recrystallized from ethanol to give compounds **5a–e**.

***5-(3-Naphthalen-2-yl-1-phenyl-1H-pyrazol-4-yl)-3-phenyl-4,5-dihydro-isoxazole (5a).***White solid, yield 81%, m.p.190–191 °C. IR (KBr) v_max_ (cm^−1^): 2967 (CH-sp^3^), 3167 (CH–Ar), 1608 (C=N). ^1^H NMR (300 MHz, DMSO-d_6_) *δ*: 3.60-3.80 (dd, 2H, isoxazoline-C4-H), 5.50 (t, *J*=11.4 Hz, 1H, isoxazoline-C5-H), 6.5 (s, 1H, pyrazole), 6.8–7.6 (m, 17H, Ar–H). ^13^C NMR (300 MHz, DMSO-d_6_) *δ*: 37.30 (isoxazoline-C4), 56.50 (isoxazoline-C5), 114.0 (pyrazole-C4), 125.0 (pyrazole-C5), 118.0–140.1 (aromatic carbons), 157.0 (pyrazole-C3), 160. 0 (isoxazoline-C3). MS (EI‏): m/z: 415 [M^+^] (17.7%). Anal. Calcd for C_28_H_21_N_3_O (415.48): C,80.94; H, 5.09; N, 10.11; Found: C,80.97; H, 5.13; N,10.23.

***3-(4-Methoxyphenyl)-5-(3-naphthalen-2-yl-1-phenyl-1H-pyrazol-4-yl)-4,5-dihydro isoxazole (5b).*** Yellow solid, yield 85%, m.p.197–198 °C. IR (KBr) v_max_ (cm^−1^): 2966 (CH-sp^3^), 3169 (CH–Ar), 1605 (C=N). ^1^H NMR (300 MHz, DMSO-d_6_) *δ*: 3.61-3.90 (dd, 2H, isoxazoline-C4-H), 3.5 (s, 3H, OCH_3_), 5.40 (t, *J*=11.5 Hz,1H, isoxazoline-C5-H), 6.6 (s,1H,pyrazole), 7.1-7.9 (m, 16H, Ar–H). ^13^C NMR (300 MHz, DMSO-d_6_) *δ*: 33.30 (isoxazoline-C4), 56.1 (OCH_3_), 60.0 (isoxazoline-C5), 115.0 (pyrazole-C4), 127.0 (pyrazole-C5), 119.0–141.0 (aromatic carbons), 158.0 (pyrazole-C3), 162.0 (isoxazoline-C3). MS (EI‏): m/z: 445 [M^+^] (18.9%). Anal. Calcd for C_29_H_23_N_3_O_2_: (445.51): C,78.18; H, 5.20; N, 9.43; Found: C,78.27; H,5.14; N,9.33.

***5-(3-Naphthalen-2-yl-1-phenyl-1H-pyrazol-4-yl)-3-p-tolyl-4,5-dihydroisoxazole (5c).*** Yellowish brown solid, yield 77%, m.p.181–182 °C. IR (KBr) v_max_ (cm^−1^): 2967 (CH-sp^3^), 3170 (CH–Ar), 1600 (C=N). ^1^H NMR (300 MHz, DMSO-d_6_) *δ*: 2.5 (s, 3H, CH_3_), 3.66-3.82 (dd, 2H, isoxazoline -C4-H), 5.55 (t, *J*=11.4 Hz, 1H, isoxazoline-C5-H), 6.7 (s, 1H, pyrazole), 7.0-7.9 (m, 16H, Ar–H). ^13^C NMR (300 MHz, DMSO-d_6_) *δ*: 20.5 (CH_3_), 32.30 (isoxazoline-C4), 60.6 (isoxazoline-C5), 115.5 (pyrazole-C4), 127.2 (pyrazole-C5), 118.0-144.0 (aromatic carbons), 157.5 (pyrazole-C3), 160.6 (isoxazoline C3). MS (EI‏): m/z: 429 [M^+^] (11.8%). Anal. Calcd for C_29_H_23_N_3_O (429.51): C,81.09; H, 5.40; N, 9.78; Found: C,81.17; H, 5.43; N,9.76.

***3-(4-Chlorophenyl)-5-(3-naphthalen-2-yl-1-phenyl-1H-pyrazol-4-yl)-4,5-dihydro isoxazole (5d).*** White solid, yield 79%, m.p.185–186 °C. IR (KBr) v_max_ (cm^−1^): 2916 (CH-sp^3^), 3046 (CH–Ar), 1594 (C=N). ^1^H NMR (300 MHz, DMSO-d_6_) *δ*: 3.31–3.50 (dd, 1H, isoxazoline-C4-H), 5.56 (t, *J*=11.6 Hz, 1H, isoxazoline-C5-H), 6.60(s, 1H, pyrazole),7-8 (m, 16H, Ar–H). ^13^C NMR (300 MHz, DMSO-d_6_) *δ*:32.5 (isoxazoline-C4), 60.8 (isoxazoline-C5), 115.6 (pyrazole-C4), 127.1 (pyrazole-C5), 117.6-140.2 (aromatic carbons), 159.7 (pyrazole-C3), 162.8(isoxazoline-C3). MS (EI‏): m/z: 449 [M^+^] (12.6%), 451 (M + 2, 4.2%). Anal. Calcd for C_28_H_20_ClN_3_ (449.93): C,74.74; H, 4,48; N, 9.34; Found: C,74.77; H,4.43; N,9.36.

***3-(3,4-Dichlorophenyl)-5-(3-naphthalen-2-yl-1-phenyl-1H-pyrazol-4-yl)-4,5-dihydro isoxazole (5e).*** White solid*, yield* 79%, m.p.187–189 °C. IR (KBr) v_max_ (cm^−1^): 2963 (CH-sp^3^), 3166 (CH–Ar), 1604 (C=N). ^1^H NMR (300 MHz, DMSO-d_6_) *δ*: 3.69 (dd, *J*=16.6,11.1 Hz, 1H, isoxazoline-C4-H), 3.82 (dd, *J*=16.7, 11.2 Hz, 1H, isoxazoline-C4-H), 6.06(t, *J*=11.6 Hz, 1H, isoxazoline-C5-H), 6.6 (s, 1H, pyrazole), 7.0-7.8 (m, 15H, Ar–H). ^13^C NMR (300 MHz, DMSO-d_6_) *δ*: 32.5 (isoxazoline-C4), 60.3 (isoxazoline-C5), 118.0 (pyrazole-C4), 128.8 (pyrazole-C5), 119.9-143.0 (aromatic carbons), 159.5 (pyrazole-C3), 162.0 (isoxazoline-C3). MS (EI‏): m/z: 484 [M^+^] (20.7%),486 (M + 2, 6.9%). Anal. Calcd for C_28_H_19_Cl_2_N_3_O (484.3): C,69.43; H,3,95; N,8.68. Found:C,69. 47; H,3.93; N,8.66.

***3’-Naphthalen-2-yl-5-aryl,1’-phenyl-3,4-dihydro-1’H-[3,4’]bipyrazolyl-2-carbo thioic acid amide (6a–e).*** To a solution of chalcones **2a–e** (1.6 mmol) in absolute ethanol (25 mL), semicarbazide hydrochloride (3.66 mmol) and piperidine (0.5 mL) were added and the solution was refluxed for 9–12 h. The reaction mixture was poured on ice water. The obtained solid was filtered off and recrystallized from ethanol to give compounds **6 a–e.**

***3′-Naphthalen-2-yl-5,1′-diphenyl-3,4-dihydro-1’H-[3,4′]bipyrazolyl-2-carbothioic acid amide (6a).*** Yellow solid, yield 65%, m.p.198–199 °C. IR (KBr) v_max_ (cm^−1^): 2966 (CH-sp^3^), 3166 (CH–Ar), 3350 (NH_2_), 1603 (C=N). ^1^H NMR (300 MHz, DMSO-d_6_) *δ*: 2.9–3.3 (dd, 2H, pyrazoline-C4-H), 5.40 (t, *J*=11.7 Hz, 1H, pyrazoline-C5-H), 6.7(s,1H, pyrazole), 7–7.7 (m, 17H, Ar–H), 10.1 (s, 2H, NH_2_, D_2_O exchangeable). ^13^C NMR (300 MHz, DMSO-d_6_) *δ*: 45.1 (pyrazoline-C4), 55.5 (pyrazoline-C5), 117.1 (pyrazole-C4), 125.1 (pyrazole-C5), 118.5–145.0 (aromatic carbons), 155.0 (pyrazoline-C3), 157.0 (pyrazole-C3), 180.0 (C=S). MS (EI‏): m/z:473 [M^+^] (16.7%). Anal. Calcd for C_29_ H_23_N_5_S (473.59): C,73.55; H, 4.90; N, 14.79. Found: C,73.47; H,4.87; N,14.73.

***5-(4-Methoxyphenyl)-3′-naphthalen-2-yl-1′-phenyl-3,4-dihydro-1’H-[3,4′] bipyrazolyl-2-carbothioic acid (6b).*** Yellow solid, yield 67%, m.p.193-194 °C. IR (KBr) v_max_ (cm^−1^): 2956 (CH-sp^3^), 3049 (CH–Ar), 3255 (NH_2_), 1597 (C=N). ^1^H NMR (300 MHz, DMSO-d_6_) *δ*: 2.8-3.03 (dd, 2H, pyrazoline-C4-H), 3.36 (s, 3H, OCH_3_), 5.66 (t, *J*=11.7 Hz, 1H, pyrazoline-C5-H), 6.0 (s, 1H, pyrazole), 6.7–7.6 (m, 16H, Ar–H), 9.66 (s, 2H, NH_2_, D_2_Oexchangeable). ^13^C NMR (300 MHz, DMSO-d_6_) *δ*:40.0 (pyrazoline-C4), 55.0 (pyrazoline-C5), 56.0 (OCH_3_), 113.9 (pyrazole-C4), 126.9 (pyrazole-C5), 118.6–148.4 (aromatic carbons), 155.6 (pyrazoline-C3), 158.3 (pyrazole-C3), 180.0 (C=S). MS(EI‏): m/z: 503 [M^+^] (12.8%). Anal. Calcd for C_30_H_25_N_5_OS: (503.62): C,71.55; H, 5.00; N, 13.91. Found: C,71.47; H,4.97; N,13.90.

***3′-Naphthalen-2-yl-1′-phenyl-5-p-tolyl-3,4-dihydro-1’H-[3,4′]bipyrazolyl-2-carbo thioic acid amide (6c).*** Yellow solid, yield 63%, m.p.184–185 °C. IR (KBr) v_max_ (cm^−1^): 2968 (CH-sp^3^), 3161 (CH–Ar), 3307 (NH_2_), 1606 (C=N). ^1^H NMR (300 MHz, DMSO-d_6_) *δ*: 2.51(s, 3H, CH_3_), 3.25–3.51 (dd, 2H, pyrazoline-C4-H), 5.43 (t, *J*=11.8 Hz, 1H, pyrazoline-C5-H), 6.7(s, 1H, pyrazole), 6.9–7.9(m, 16H, Ar–H), 10.5 (s,1H, NH_2_, D_2_Oexchangeable). ^13^C NMR (300 MHz, DMSO-d_6_) *δ*: 20.6 (CH_3_), 45.5 (pyrazoline-C4), 56.6 (pyrazoline-C5), 117.5 (pyrazole-C4), 125.5 (pyrazole-C5), 118.7–144.0 (aromatic carbons), 155.6 (pyrazoline-C3), 157.50 (pyrazole-C3), 180.2 (C=S). MS(EI‏): m/z: 487 [M^+^] (16.3%). Anal. Calcd for C_30_H_25_N_5_S (487.62): C,73.89; H, 5.17; N, 14.36. Found: C,73.87; H,5.13; N,14.43.

***5-(4-Chlorophenyl)-3′-naphthalen-2-yl-1′-phenyl-3,4-dihydro-1’H-[3,4′] bipyrazo lyl-2-carbothioic acid (6d).*** Yellow solid, yield 69%, m.p.186–187 °C. IR (KBr) v_max_ (cm^−1^): 2963 (CH-sp^3^), 3162 (CH–Ar), 3310 (NH_2_), 1606 (C=N). ^1^H NMR (300 MHz, DMSO-d_6_) *δ*: 2.90–3.37 (dd, 1H, pyrazoline-C4-H), 5.62 (t, *J*=11.4 Hz, 1H, pyrazoline-C5-H), 6.6 (s, 1H, pyrazole), 7.0–7.8 (m, 16H, Ar–H), 10.1 (s, 1H, NH_2_, D_2_O exchangeable). ^13^C NMR (300 MHz, DMSO-d_6_) *δ*: 45.1 (pyrazoline-C4), 56.6 (pyrazoline-C5), 117.6.0 (pyrazole-C4), 125.3 (pyrazole-C5), 118.9–143.0 (aromatic carbons), 157.7 (pyrazoline-C3), 160.0 (pyrazole-C3), 180.0 (C=S). MS (EI‏): m/z: 507 [M^+^] (20%), 509 (M + 2, 6.7%). Anal. Calcd for C_29_H_22_ClN_5_S (508.3): C,68.56; H, 4.36; N, 13.79. Found: C, 68.57; H,4.33; N,13.63.

***5-(3,4-Dichlorophenyl)-3′-naphthalen-2-yl-1′-phenyl-3,4-dihydro-1’H-[3,4′]bi pyrazolyl-2-carbothioic acid (6e)*.** Yellow solid, yield 69%, m.p. 180–181 °C. IR (KBr) v_max_ (cm^−1^): 2967 (CH–sp^3^), 3163 (CH–Ar), 3317 (NH_2_), 1600 (C=N). ^1^H NMR (300 MHz, DMSO-d_6_) *δ*: 3.01 (dd, *J*=16.1, 11.2 Hz, 1H, pyrazoline-C4-H), 3.6 (dd, *J*=16.2, 11.2 Hz, 1H, pyrazoline-C4-H), 5.25 (t, *J =* 11.5 Hz, 1H, pyrazoline-C5-H), 6.8 (s, 1H, pyrazole), 7.0–7.8 (m, 15H, Ar–H), 10.2 (s, 1H, NH_2_, D_2_O exchangeable). ^13^C NMR (300 MHz, DMSO-d_6_) *δ*: 45.3 (pyrazoline-C4), 56.3 (pyrazoline-C5), 117.3.0 (pyrazole-C4), 125.4 (pyrazole-C5), 118.1–142.0 (aromatic carbons), 155.3 (pyrazoline-C3), 157.1 (pyrazole-C3), 180.0 (C=S). MS (EI‏): m/z: 541 [M^+^] (22.1%), 543 (M + 2, 7.3%). Anal. Calcd for C_29_H_21_Cl_2_N_5_S (542.48):C,64.21; H, 3.90; N,12.91. Found: C,64.27; H,3.83; N,12.83.

### 2.3. In vitro assays for biological antioxidant Activity

***Chemicals***: All chemicals required for all assays were used as analytical grade, and were purchased from Sigma-Aldrich Chemicals Co., St. Louis, MO, USA.

#### 2.3.1. DPPH scavenging method

The DPPH scavenging activity of all synthesized compounds was measured as previously described by Nahar et al.^48^ with some modifications. Briefly, 100 µL of different concentrations of the tested compounds (12.5, 25, 50, 100, and 200 µg/mL) were pipetted into a 96-well plate. Then, 100 µL of 100-µM DPPH methanolic solution were added to each well and the plate was incubated protected from light at room temperature for 30 min. The absorbance of the solution was measured at λ_517 nm._^49^ Ascorbic acid was used as the positive control while DMSO was the negative control. The percentage of DPPH scavenging activity was calculated according to the following equation:

$$\%\;\text{of}\;\text{DPPH}\;\text{scavenging}=[(A_{\text{control}}–A_{\text{sample}})/A_{\text{control}}]\;\times 100,$$

where *A*_control_ is the absorbance of the control reaction (with all reagents except the test compound), and *A*_sample_ is the absorbance of the test sample. Linear regression analysis was performed to calculate drug concentration showing 50% free radical inhibition activity (IC_50_). All tests were performed in triplicates.

#### 2.3.2. No scavenging method

The NO scavenging activity of all synthesized compounds was measured as previously described by Ho et al.^50^ Briefly, 50 µL of different concentrations of the tested compounds (12.5, 25, 50,100, and 200 µg/mL) were pipetted into a 96-well plate. Then, 50 µL of 10-mM sodium nitroprussides dissolved in phosphate-buffered saline PBS (pH 7.4) were added to each well and the plate was incubated for 90 min at room temperature. Next, an equal volume of Griess reagent (1% of sulphanilamide and 0.1% of naphthyl ethylene diamine in 2.5% H_3_PO_3_) was added to each well to measure the nitrite content. The absorbance of the formed pink-coloured chromophore was measured at λ _546 nm_. DMSO and ascorbic acid were used as the negative and positive control, respectively. All tests were performed in triplicate. The percentage of NO scavenging activity was calculated according to the following equation:

$$\%\;\text{of}\;\text{NO}\;\text{scavenging}=\left[\left(A_{\text{control}}-A_{\text{sample}}\right)/A_{\text{control}}\right]\times 100$$

where *A*_control_ is the absorbance of the control reaction (with all reagents except the test compound), and *A*_sample_ is the absorbance of the test sample. Linear regression analysis was performed to calculate drug concentration showing 50% free radical inhibition activity (IC_50_).

#### 2.3.3. Superoxide scavenging assay (O_2_^−.^)

The improved pyrogallol autoxidation method was used to determine O_2_^−.^ RSA of all synthesized compounds as previously described^51^. Briefly, 50 µL of test compounds (12.5, 25, 50,100, and 200 µg/mL) was added to 2900 µl of 5-mM Tris HCl buffer (0.05 M, pH 7.4) containing 1-mM Na_2_EDTA. Next, 50 µL of 60-mM pyrogallol in1mM HCl had been thoroughly mixed with the mixture. The absorbance of the reaction mixture was measured at A_325_ nm every 30 s for 5 min. O_2_^−^ RSA was expressed by the oxidation degree of a test group in comparison to that of the control. The absorbance at 325 nm was measured against the Tris-HCl buffer every 30 s for 5 min. The percentage of scavenging effect was calculated using the following equation:

$$O_{2}{}^{-}\;\text{radical}\;\text{scavenging}\;\%=\left[\left(\Delta A_{325,\;\text{control}}/T\right)-\left(\Delta A_{325\text{nm},\;\text{sample}}/T\right)/\Delta A_{325\text{nm},\;\text{control}}/T\right]\times 100$$

where Δ_A325__nm, control_ is the increase in A_325 nm_ of the reaction mixture without the sample and ΔA_325 nm, sample_ is that for the mixture with the sample; T=5 min. The experiments were performed in triplicate. The IC_50_ value was defined as the concentration for 50% superoxide free radical inhibition and was calculated by linear regression.

### 2.4. In-vitro lipoxygenase inhibition activity

The assay was performed using Cayman’s lipoxygenase inhibitor screening assay kit (Catalog No. 760700, Cayman Chemical, USA) according to the manufacturer’s instructions. Briefly, 90 µL of 15-LOX was pipetted into a 96-well plate. Next, 10 µL of test compound at concentrations (2.5, 5.0 and 10 µM) dissolved in DMSO were added to each well. The reaction was initiated by adding 10 µL substrate (AA) and the plate was placed on a shaker for at least 5 minutes. Finally, 100 µL of chromogen (prepared according to manufacturer’s instructions) was added to each well to stop enzyme catalysis and develop the reaction. 100 µL of Assay buffer (0.1 M Tris-HCl, pH7.4) was used in blank wells. Quercetin and DMSO were used as the positive control and 100% initial activity, respectively. The absorbance of the solution was measured at λ 490–500 nm. The percentage inhibition was calculated according to the following equation:

$$\%\;\text{inhibition}\;=\left[\left(\text{IA}-A_{\text{inhibitor}\;\text{sample}}\right)/\text{IA}\right]\times 100$$

where (IA) is the 100% initial activity and (*A*_inhibitor sample)_ is the absorbance of the test sample. Dose–response curve was plotted between % inhibition and the drug concentration. The non-linear dose–response curve was used for calculating drug concentration showing 50% enzyme inhibition.

### 2.5. In vivo biological antioxidant assays

To determine *in vivo* antioxidant potentials of the test compounds which showed promising *in vitro* antioxidant activities, CAT activity, GSH and MDA levels were assayed in liver of treated animals.

#### 2.5.1. Animals

The complete progress of the experiment was conducted using male Wistar albino rats (200–250 g), delivered by the Institutional Breeding House, Egypt, reared and maintained in the animal house of the institution. The animals had free access to food and water *ad libitum* and maintained in a controlled environment under standard conditions of temperature and humidity with an alternating 12 h light and dark cycle for about a week for acclimatization. The protocol of the study was approved by the Animal Ethics Committee of the Faculty of Pharmacy, Helwan University (ethical code number: 05A2019; date: October 2019). The study was conducted in accordance with the EC, directive 86/609/EEC for animal experiments.

#### 2.5.2. Acute oral toxicity study

The acute toxicity study of the selected compounds was performed on albino rats according Organization for Economic Co-operation and development guidelines-425^52^. The animals were fasted overnight prior to the experiment with free access to water. Selected drugs were administered at doses equal to and half of Ascorbic Acid dose (50 and 100 mg/kg/p.o.), and the behavioural change was observed up to 24 h. The selected compounds were found to be non-toxic in the selected doses. Dose selected for *in vivo* antioxidant study was 100 mg/kg B.W.

#### 2.5.3. Animal treatment

Rats are weighed at the beginning and at the end of experiment. Fifty-four male albino rats (*n*=6) were divided into nine different groups. Group I served as a control group and treated with the same volume vehicle only. Group II treated with 100 mg/kg of Ascorbic acid as standard antioxidant drug. Groups (III–IX) orally administered 100 mg/kg of compounds **3a, 4e, 5b, 5c, 6a, 6c, 6e**, respectively, for 3 days. The animals were sacrificed by cervical dislocation 24 h after the last dose. Sacrificing is carried out at the same time of the day, to avoid the circadian variation in the level of tissue GSH^53^. Each liver was excised, weighed, rinsed in ice-cold normal saline and frozen for not more than 72 h await analysis of endogenous antioxidant status (GSH levels and CAT activity) and lipid peroxide concentrations. For performing biochemical assays, a 10% liver homogenate in 10 mM phosphate buffer was prepared using tissue homogeniser (Glas-Col^®^, Cat no.099C K6424, TERRE HAUTE, USA).

##### 2.5.3.1. CAT activity

Catalase activity in 10% liver homogenates was determined spectrophotometrically according to Sinha AK^54^. The decrease in absorbance at 240 nm due to H_2_O_2_ decomposition was measured and the results were expressed in U/mg tissue.

##### 2.5.3.2. Determination of reduced glutathione

Levels of glutathione (GSH) in liver homogenates were assayed by the deproteinization of tissue homogenate^55^. Then 200 mL supernatant was mixed with di-potassium hydrogen phosphate buffer (pH 8) and 0.4% 5,5′-dithiobis-2-nitrobenzoic acid (Ellman’s reagent). The yellow-coloured substance formed was measured at 412 nm. The results were expressed as GSH mg/g tissue.

##### 2.5.3.3. Determination of lipid peroxide level

Lipid peroxidation level in the liver homogenates was determined as thiobarbituric acid reactive substances (TBARS) by measuring malondialdehyde ‘MDA’ level according to Mihara and Uchiyama^56^. Briefly, 0.5 mL supernatant of tissue homogenate was mixed with 0.6% thiobarbituric acid (TBA) and 1% orthophosphoric acid solution, and heated in a boiling water bath for 45 min. The pink-coloured chromogen formed by the reaction of TBA with MDA was extracted by n-butanol and measured at 535 nm. The results were expressed as nmol/g tissue.

### 2.6. Data presentation and statistical analysis

The data were represented as mean ± SEM. Significant differences between groups were tested by using GraphPad InStat software version 3.05 (GraphPad Inc., La Jolla, CA, USA). Appropriate graphs were plotted when needed using GraphPad Prism version 5 for Windows (GraphPad Inc., USA). The results were analysed using one-way analysis of variance (ANOVA) with *post hoc* Scheffe’s test. A value of *p* < 0.05 was considered statistically significant.

### 2.7. Molecular modelling procedure

The modelling experiment described in this study was performed by using the Discovery Studio (DS) version 4.5 (Accelrys Inc., San Diego, CA, USA) software^57^. The required pdb coordinates were downloaded from the Brookhaven website (www.rcsb.org). The hydrogen atoms were then added to both the small molecule and the 15-LOX enzyme structure. The atom and bond types as well as the protonation state for the small molecule and the binding site were checked and corrected when needed. Water molecules were deleted. This was followed by minimising the complex with the DS force field using the default parameters. The resulted binding mode of the designed compound in bound to catalytic active site of 15-LOX will be discussed later.

## 3. Results and discussion

### 3.1. Chemistry

**Scheme 1.**
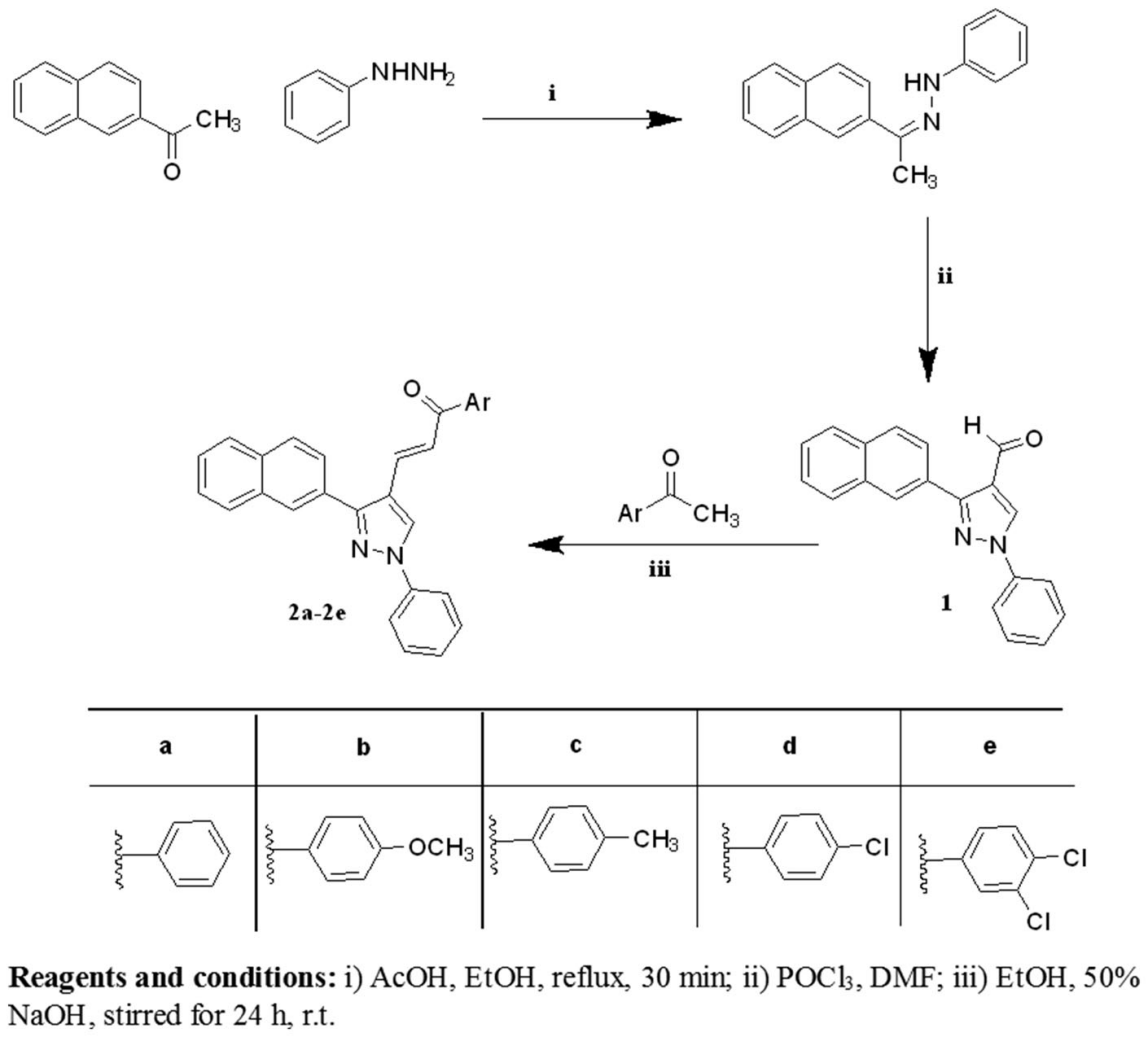
Synthesis of the designed compounds **1** and **2 (a–e).**

**Scheme 2.**
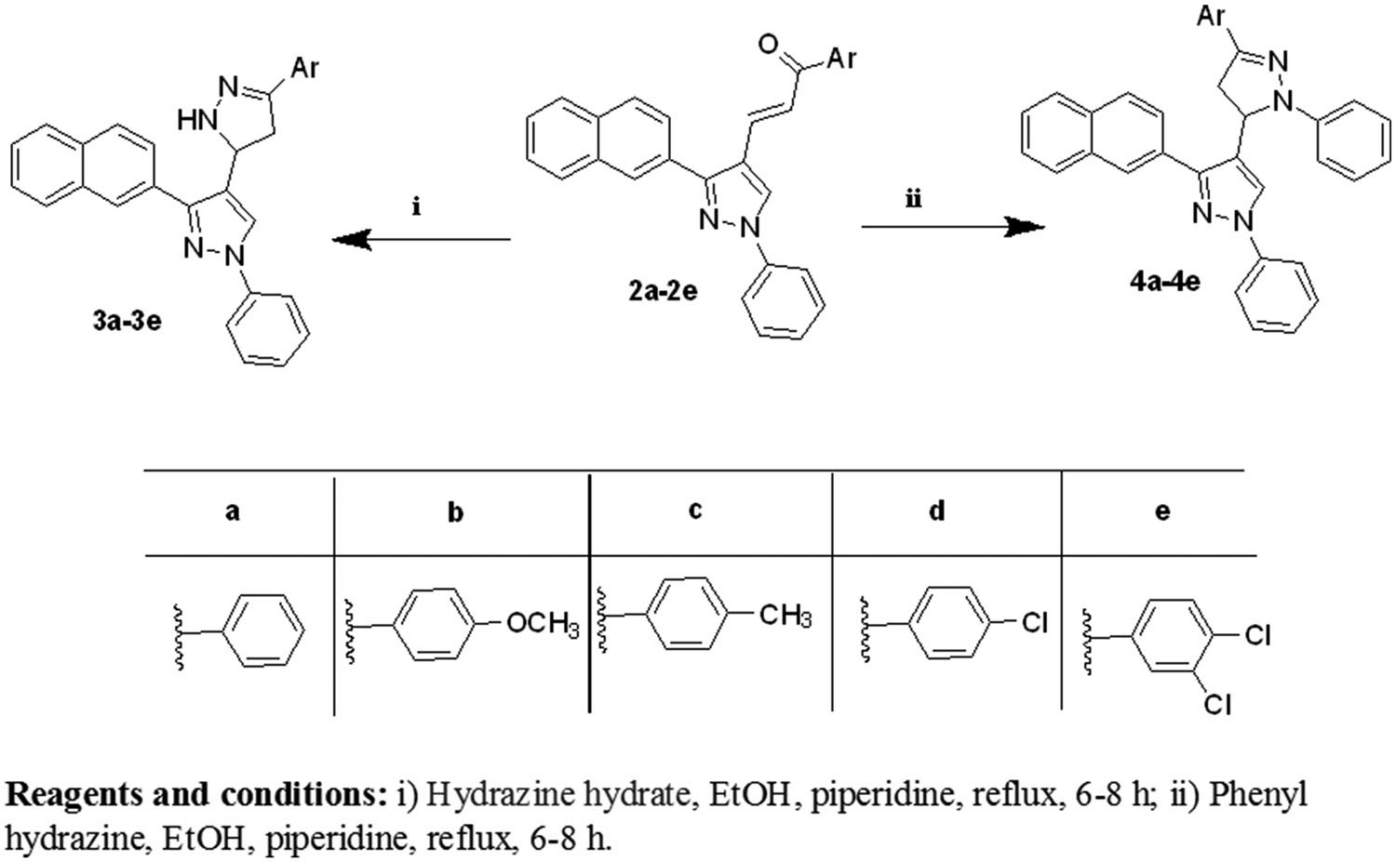
Synthesis of the designed compounds **3 (a–e)** and **4 (a–e).**

**Scheme 3.**
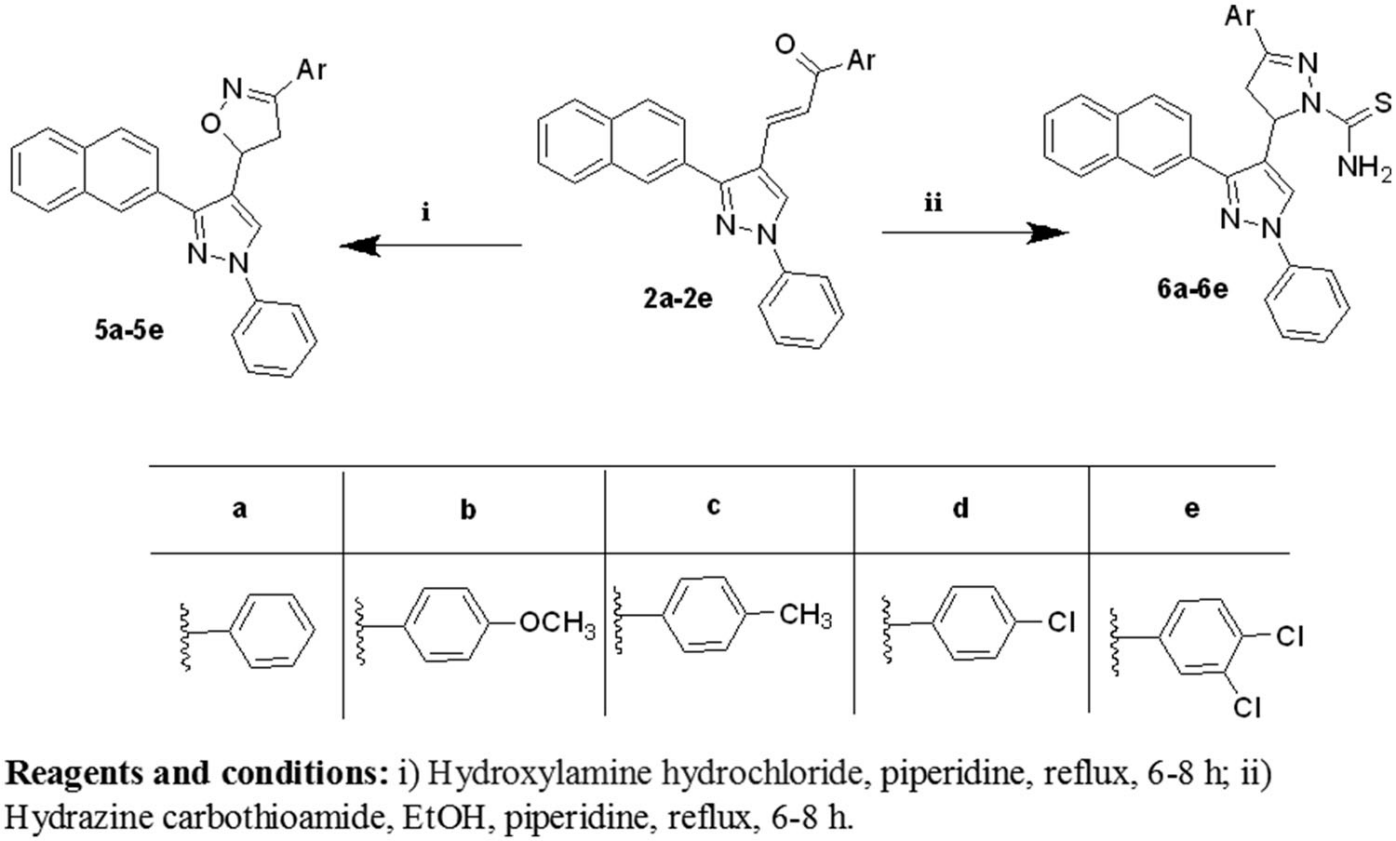
Synthesis of the designed compounds **5 (a–e)** and **6 (a–e).**

**Figure 4.**
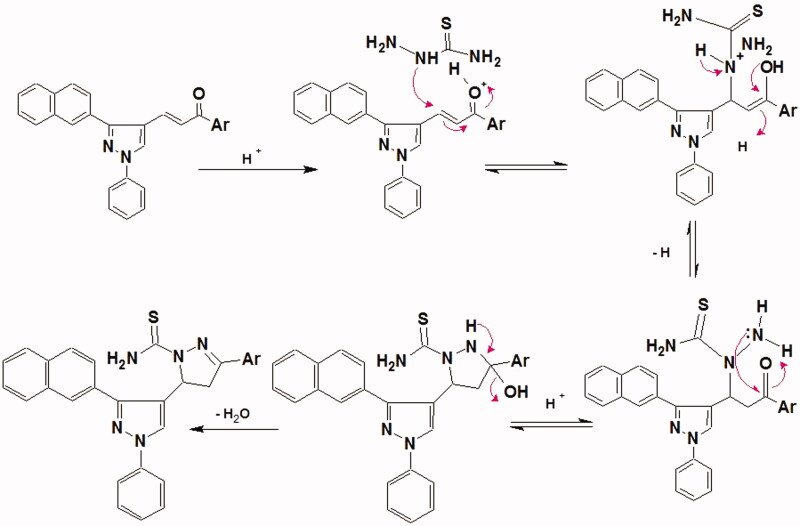
Proposed reaction mechanism for the formation of pyrazole-carbothioamide.

The synthesis of the target compounds **(2-6)** was depicted in Schemes (1-3). The key starting derivative 3-(2-naphthyl)-1-phenyl-1H-pyrazole-4-carbaldehyde (**1**) was prepared via Vilsmeier-Haack reaction^14^ of a phenyl hydrazone, derived from the reaction of β-acetyl naphthalene with phenyl hydrazine, in refluxing absolute ethanol containing few glacial acetic acid followed by the addition of two equivalents of dimethyl formamide and POCl_3_. Claisen-Schmidt condensation^58^ of (**1)** with different aromatic ketones such as 1-phenylethanone, 1-(4-methoxyphenyl)ethanone, 1-(4-methylphenyl)ethanone, 1-(4-chlorophenyl)ethanone and 1-(3,4-dichloro phenyl)ethanone was performed in 30% ethanolic sodium hydroxide solution at room temperature to afford the corresponding chalcones **(2a–e)**, respectively, as outlined in (Scheme 1) . The formed chalcone derivatives **(2a–e)** was used as key intermediates for synthesizing the target pyrazole-pyrazolines **(3)** and **(4)** through 1,4-addition of hydrazine hydrate or phenyl hydrazine to the α, β-unsaturated carbonyl system of the precursor chalcones **2a–e**, followed by dehydration and rearrangement. Cyclocondensation of the chalcones **(2a–e)** with hydrazine hydrate or phenyl hydrazine^59–61^ in absolute ethanol with catalytic amount of piperidine gave pyrazolines **(3a–e)** and phenylpyrazolines **(4a–e),** respectively (Scheme 2). Cyclization of chalcones **(2a–e**) into the corresponding isoxazolines (**5a–e**) was conducted by condensation of the chalcones with hydroxylamine hydrochloride^62^ in ethanol containing a catalytic amount of piperidine to give the target derivatives. In addition, pyrazoline-1-carboxamides **(6a–e)** were prepared by base-catalysed cyclization of chalcones **2a–e** through reaction with semicarbazide HCl^63^ in absolute ethanol and piperidine (Scheme 3). The reaction mechanism for formation of carbothioamide is via the nucleophilic attack of thiosemicarbazide at β-carbon of the α-β unsaturated C=O of chalcone followed by the proton transfer and intramolecular cyclization of molecule by the nucleophilic attack of NH_2_ to carbonyl carbon which is stabilized by the proton transfer and further dehydration leads to the formation of pyrazoline (Figure 4).

The formed new compounds (**2-6)** were confirmed by IR, ^1^HNMR, ^13^C NMR, mass spectroscopy and microanalysis. The IR spectrum of compounds **2a–e** exhibited characteristic bands at around 1690 cm^−1^ for C=O. ^1^H NMR spectra showed two doublets signals at δ 6.2–7.4 ppm for CH=CH protons of chalcones. The ^1^H NMR spectra of compounds **3-6** showed signal doublet of doublet at *δ* 2.80–3.90 ppm and triplet at *δ* 5.20-5.66 ppm corresponding to the protons at C-4 and C-5 of the pyrazoline ring in addition to the signals of pyrazole and other protons (CH_3_, OCH_3_, and aromatic Hs) . Moreover, the ^1^H NMR spectra of compounds **3** and **6** showed singlet exchangeable signal at around *δ* 8 ppm or δ 10 ppm corresponding to (NH) of the pyrazoline or (NH_2_) of the carboxamide, respectively. ^13^C NMR showed the characteristic signals at d *δ* 32.1-38.3 and 55.1-60.6 ppm corresponding to C-4 and C-5 carbon of the pyrazoline ring, respectively. In addition to the other signals for the carbons of the target compounds, The ^13^C NMR spectra of compounds **2** and **6** showed the presence of signals corresponding to C=O around at *δ* 187 ppm and C=S at around *δ* 180 ppm, respectively (cf. experimental part).

### 3.2. Biological antioxidant studies

This study presents the synthesis and biological evaluation of antioxidant activity of compounds having pyrazole, naphthalene and pyrazoline/isoxazoline pharmacophore. Most of LOX inhibitors show antioxidant or free radical scavenging activities as lipoxygenation occurs via a carbon centred radical^64^. Thus, all compounds **2–6 (a–e)** were investigated for their RSA by DPPH, NO and superoxide assays while Ascorbic Acid (AA) was used as antioxidant reference standard. The *in vitro* antioxidant activities of tested compounds were expressed as IC_50_ values (Table 1).

**Table 1.** *In vitro* antioxidant potential and 15-LOX inhibition activity of compounds (2–6).

Compounds	DPPH IC_50_^a^ (µg/mL)	NO IC_50_^a^ (µg/mL)	Superoxide IC_50_^a^ (µg/mL)	15-LOX IC_50_^a^ (µM)
**2a**	>200	>200	>200	ND^b^
**2b**	182.17 ± 0.99	78.25 ± 1.25	>200	3.13 ± 0.09
**2c**	192.70 ± 1.63	>200	>200	2.80 ± 0.06
**2d**	>200	>200	>200	ND
**2e**	118.99 ± 1.78	11.04 ± 0.72	>200	4.63 ± 0.09
**3a**	13.99 ± 0.78	27.65 ± 1.53	50.42 ± 1.45	2.23 ± 0.07
**3b**	11.70 ± 0.29	147.95 ± 1.32	118.65 ± 1.03	4.60 ± 0.06
**3c**	12.06 ± 1.17	>200	>200	3.77 ± 0.07
**3d**	>200	>200	>200	ND
**3e**	9.63 ± 0.55	175.72 ± 1.41	129.12 ± 0.82	2.53 ± 0.07
**4a**	34.39 ± 1.03	>200	176.14 ± 1.63	2.53 ± 0.09
**4b**	21.28 ± 1.14	>200	65.63 ± 1.46	4.00 ± 0.06
**4c**	24.42 ± 0.9	179.9 ± 1.31	145.7 ± 1.42	1.83 ± 0.07
**4d**	>200	>200	>200	ND
**4e**	19.56 ± 1.06	57.01 ± 1.29	130.19 ± 1.1	3.53 ± 0.07
**5a**	97.17 ± 1.4	37.5 ± 1.36	192.37 ± 1.74	5.53 ± 0.07
**5b**	46.62 ± 1.63	75.53 ± 1.43	101.8 ± 1.39	4.37 ± 0.09
**5c**	47.27 ± 1.13	38.99 ± 1.31	44.54 ± 1.44	4.23 ± 0.07
**5d**	>200	>200	>200	ND
**5e**	38.44 ± 1.28	96.56 ± 1.4	168.77 ± 1.42	3.00 ± 0.06
**6a**	20.47 ± 1.43	36.37 ± 0.75	127.25 ± 1.47	1.50 ± 0.06
**6b**	12.02 ± 0.63	198.08 ± 1.28	>200	1.90 ± 0.06
**6c**	18.98 ± 1.73	23.79 ± 0.83	140.17 ± 1.52	2.10 ± 0.06
**6d**	>200	148.08 ± 1.36	>200	1.67 ± 0.03
**6e**	9.66 ± 0.34	71.39 ± 1.25	87.31 ± 1.58	1.57 ± 0.03
**Ascorbic acid**	13.67 ± 0.97	37.9 ± 1.31	124.99 ± 1.32	2.5
**Quercetin**	ND	ND	ND	3.34

^a^ IC_50_ values are expressed as a mean ± SEM of three experiments.

^b^ Not determined.

#### 3.2.1. DPPH scavenging activity

**Figure 5.**
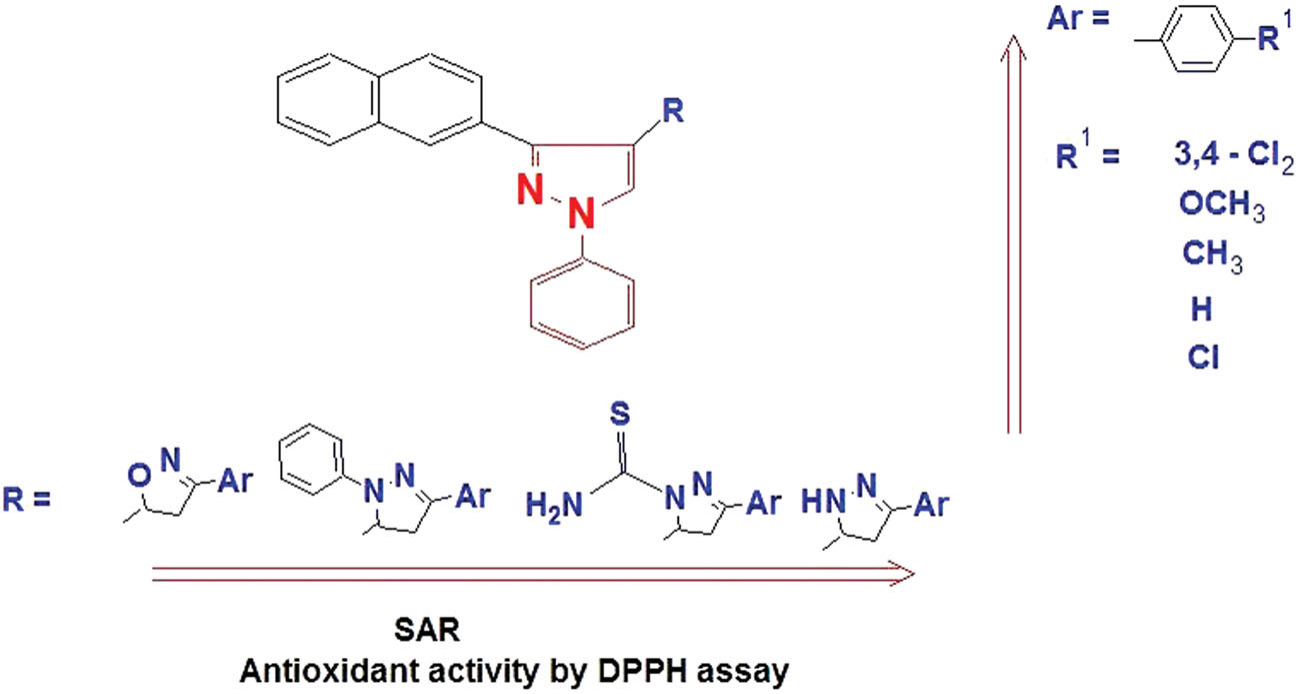
Structure activity relationship of the pyrazole derivatives against DPPH radical scavenging assay.

Currently, antioxidants showing DPPH scavenging activity are receiving attention due to their role as anticancer, anti-inflammatory and antiaging agents^65^. Therefore, the antioxidant potential of all novel molecules **(2–6)** was determined using DPPH radical scavenging assay in comparison with ascorbic acid (AA) as control treatment. The mechanism of radical scavenging is based on acidic H-atom transfer from the compound to the DPPH to form DPPH-H. The results are presented in Table 1. Out of the twenty-five tested pyrazole derivatives, sixteen showed moderate to potent activity, which indicates their radical scavenging and their reducing activities. The rest being less active derivatives .The pyrazolyl pyrazolines **(3a-d)** and pyrazoline carbothioamides **(6a-d)** have potent antioxidant activities while pyrazolyl isoxazolines **(5a-d)** have demonstrated moderate RSA. The most active compounds were **3b, 3c, 3e, 6b** and **6e** (IC_50_=11.70 ± 0.29, 12.06 ± 1.17, 9.63 ± 0.55, 12.02 ± 0.63, and 9.66 ± 0.34 μg/mL, respectively). They exhibited potent RSA than ascorbic acid (IC_50_= 13.67 ± 0.97 μg/mL). Suggesting that, the presence of free NH of pyrazoline enhances the antioxidant activity by increasing their hydrogen donor capacity. Moreover, good radical scavenger property of S atom C=S and free NH_2_ that act as hydrogen donor in pyrazoline carbothioamides possessed potent DPPH RSA. Compounds **3a**, **6a,** and **6c** (IC_50_=13.99 ± 0.78, 20.47 ± 1.43 and 18.98 ± 1.73 μg/mL, respectively) displayed good RSA but lower than ascorbic acid. In addition, *N*-phenyl pyrazolyl pyrazolines **4b** (IC_50_=21.28 ± 1.14 μg/mL), **4c** (IC_50_=24.42 ± 0.9 μg/mL) and **4e** (IC_50_= 19.56 ± 1.06 μg/mL) showed good activity in comparison to standard treatment **AA**. The lower DPPH RSA of *N*-phenyl pyrazolyl pyrazolines **(4a–e)** and pyrazolyl isoxazolines **(5a–e)** prove the significant role of NH of pyrazoline in antioxidant activity. All compounds **(3–6)** showed higher antioxidant RSA than their precursors, chalcones **(2a–e)** thus indicating that pyrazoline and isoxazoline rings enhance RSA of these compounds. SAR studies showed that antioxidant activity of compounds tested by DPPH assay depends not only on the type of heterocyclic pharmacophore but also on substituents R on the aromatic ring of pyrazoline/isooxazoline since antioxidant activity is related to electron or hydrogen donation capacity to DPPH**^.^**Radicals (Figure 5). Regarding heterocyclic pharmacophore, the order of free radical scavenging activity (FRSA) were found to be: (**3e** > **6e**> **4e**> **5e**). Pyrazolines **3** and pyrazoline carbothioamides **6** have higher FRSA than *N*-phenyl pyrazolines **4** and isoxazolines **5**. For compounds **3e** and **6e** their potent antioxidant activity is due to the presence of pyrazoline and carbothioamide moiety^30^^,^^66^. While the replacement of N atom by O atom gives lower antioxidant activity as shown in isoxazolines **5**. Concerning substitution patterns of pyrazolineAQ4/isoxazoline. On the other hand, the order of antioxidant activity of pyrazoline and isoxazoline compounds was found to be: 3,4-(Cl) _2_ **(3e)** > 4-OCH_3_ **(3b)** > 4-CH_3_ **(3c)** > H **(3a)** > 4-Cl **(3d)**, in descending order.

The di-halogenated compounds exhibited significant DPPH RSA than the corresponding chloro-substituted compounds. Pyrazole derivative **3e** and **6e** show the most potent antioxidant activity, both having 3,4 (di-Cl) substituents on phenyl ring, which is in accordance with the reported results^67^. Presence of electron donating groups such as OCH_3_ and CH_3_ are more beneficial than unsubstituted or mono chloro-substituted phenyl ring, which may be due to + I and mesomeric effects^68^. This all indicate that the physicochemical properties of the designed compound impose an important role in the extent of its antioxidant activity. It is notable that the calculated cLog P for these derivatives are high (more lipophilic) compared to the standard treatment. In addition, these derivatives are cyclized heterocyclic analogues with fewer rotatable bonds that make them more favourable for cellular permeability compared to the standard treatment. It was found that the more lipophilic is the compound, the more active it is as 15 LOX-inhibitor. Also, the more electron withdrawal substitutions on the aromatic side chain of the heterocyclic ring, the more antioxidant activity was observed.

#### 3.2.2. No scavenging activity

NO assay was used to determine the scavenging power of the target compounds **(2–6)** to NO radical. The results are presented in Table 1. Sodium nitroprusside in aqueous solution at physiological pH spontaneously generates Nitrite oxide which interacts with oxygen to produce Nitrite ions, which can be measured at 550 nm by spectrophotometer in the presence of Griess reagent^69^. Compounds **2e, 6c, 3a** and **6a** showed NO scavenging activity through competing with oxygen to scavenge for the nitrite radical; higher than that of ascorbic acid (IC_50_=11.04 ± 0.72, 23.79 ± 0.83, 27.65 ± 1.53 and 36.37 ± 0.75 μg/mL, respectively). Pyrazole **2e** was the most potent antioxidant derivative via reducing nitrite production with 3.4 folds that of ascorbic acid, Pyrazolyl isoxazolines **5a, 5c** (IC_50_=37.5 ± 1.36 and 38.99 ± 1.31 μg/mL, respectively) displayed comparable potency to ascorbic acid. Moreover, *N*-phenyl pyrazolyl pyrazoline **4e** displayed good antioxidant activity (IC_50_=57.01 ± 1.29 μg/mL). SAR studies showed that pyrazoline carbothioamide **6c** and pyrazolyl pyrazoline **3a** exhibited higher NO scavenging activity than pyrazolyl isoxazoline **5a** and *N*-phenyl pyrazolyl pyrazoline **4e**. This confirms that pyrazoline and carbothioamide rings were favourable substitutions over isoxazoline and *N*-phenyl pyrazoline for the antioxidant activity of the tested compounds against NO assay.

#### 3.2.3. Superoxide scavenging activity

All target compounds **(2–6)** were evaluated for their antioxidant activity via superoxide scavenging assay to estimate their capability to scavenge O_2_^−^^.^ and so, preventing the formation of elemental oxygen. The resulted IC_50_ values were measured in μg/mL as shown in Table 1. Compounds **5c, 3a, 4b, 6e** and **5b** (IC_50_=44.54 ± 1.44, 50.42 ± 1.45, 65.63 ± 1.46, 87.31 ± 1.58 and 101.8 ± 1.39 μg/mL, respectively) possessed pronouncing superoxide scavenging activity than ascorbic acid (IC_50_=124.99 ± 1.32 μg/mL). Pyrazolyl isoxazoline **5c** and pyrazolyl pyrazoline **3a** were the most potent derivatives with 2.8 and 2.5 folds of ascorbic acid, respectively. In addition, pyrazolyl pyrazolines **3b** and **3e** (IC_50_=118.65 ± 1.03 and 129.12 ± 0.82 μg/mL, respectively), pyrazoline carbothioamide **6a** (IC_50_= 127.25 ± 1.47 μg/mL) displayed comparable O2**^−.^** scavenging potency to ascorbic acid. Moreover, pyrazoline carbothioamide **6c** (IC_50_ = 140.17 ± 1.52 μg/mL), *N*-phenylpyrazoline **4c, 4e** (IC_50_= 145.7 ± 1.42 and 130.19 ± 1.1 μg/mL, respectively) showed good superoxide scavenging activity. SAR studies showed that most of synthesized compounds revealed higher activity than the parent chalcones in superoxide scavenging assay. Pyrazoline **3a**, isoxazoline **5c**, pyrazoline carbothioamide **6e** and *N*-phenylpyrazoline **4b** rings showed a significant RSA towards superoxide radical anion. Di-halogenated compounds displayed good superoxide RSA while, mono halogenated ones didn^’^t.

### 3.3. In vitro 15-lipoxygenase inhibition activity

All target compounds (**2–6**) were tested against Soybean 15-LOX enzyme. The results are expressed as IC_50_ values (µM) as shown in Table 1. Compounds **2b, 2c, 3a, 3e, 4a, 4c, 5e, 6a, 6b, 6c** and **6d** showed potential 15-LOX inhibition activity when compared to quercetin (IC_50_=3.34 µM) as reference inhibitor. Carbothioamides **6a, 6e** in which the pyrazoline ring is substituted with phenyl moiety and 3,4-di-Cl phenyl, were the most potent compounds (IC_50_=1.50 ± 0.06 and 1.57 ± 0.03 µM, respectively) with 2.2 and 2.1 folds that of quercetin, respectively. Pyrazoline **3c** and *N*-phenyl pyrazoline **4e** showed comparable potency to that of quercetin (IC_50_= 3.77 ± 0.07 and 3.53 ± 0.07 µM, respectively). Moreover, pyrazole **2e** (IC_50_=4.63 ± 0.09 µM), pyrazoline **3b** (IC_50_=4.60 ± 0.06 µM), isoxazolines **5a, 5b,** and **5c** (IC_50_= 5.53 ± 0.07, 4.37 ± 0.09 and 4.23 ± 0.07 µM, respectively) and *N*-phenyl pyrazoline **4b** (IC_50_=4.00 ± 0.06 µM) displayed good 15-LOX inhibitory activity but lower than quercetin. The results of the tested compounds **(2–6)** as 15-LOX inhibitors emphasized the important role of 3-naphthylpyarazole in this enzymatic assay regardless the derivative was either α, β-unsaturated ketone **2c**, pyrazoline **3a,** *N*-phenylpyrazoline **4c,** isoxazoline **5e or** pyrazoline carbothioamide counterpart **6a**. Those derivatives were superior to quercetin in 15-LOX inhibition. 15-LOX inhibition appeared to be **(6a> 4c >3a> 2c> 5e)** to confirm the excel pyrazoline carbothioamide over pyrazoline, *N*-phenyl pyrazoline and isoxazoline for antioxidant activity of the tested compounds against Soybean 15-LOX enzyme. It was also noticed that di-halogenated derivatives showed significant 15-LOX inhibition activity. This might be due to better fitting of derivative into the catalytic pocket of 15-LOX enzyme. In summary, compounds **3a, 4e, 5b, 5c, 6a, 6c** and **6e** showed significant RSA in all three methods in comparison with ascorbic acid and 15-LOX inhibition potency using quercetin as standard. This suggests an important influence of EDGs (CH_3_, OCH_3_) and di-halogen (di-Cl) in benzene ring. Regarding heterocyclic pharmacophore, pyrazoline carbothioamide and pyrazoline showed higher RSA and 15-LOX inhibition potency than *N*-phenyl pyrazoline and isoxazoline and these observations should be regarded in the future on the designed LOX inhibitors.

### 3.4. In vivo estimation of antioxidant activity

**Figure 6.**
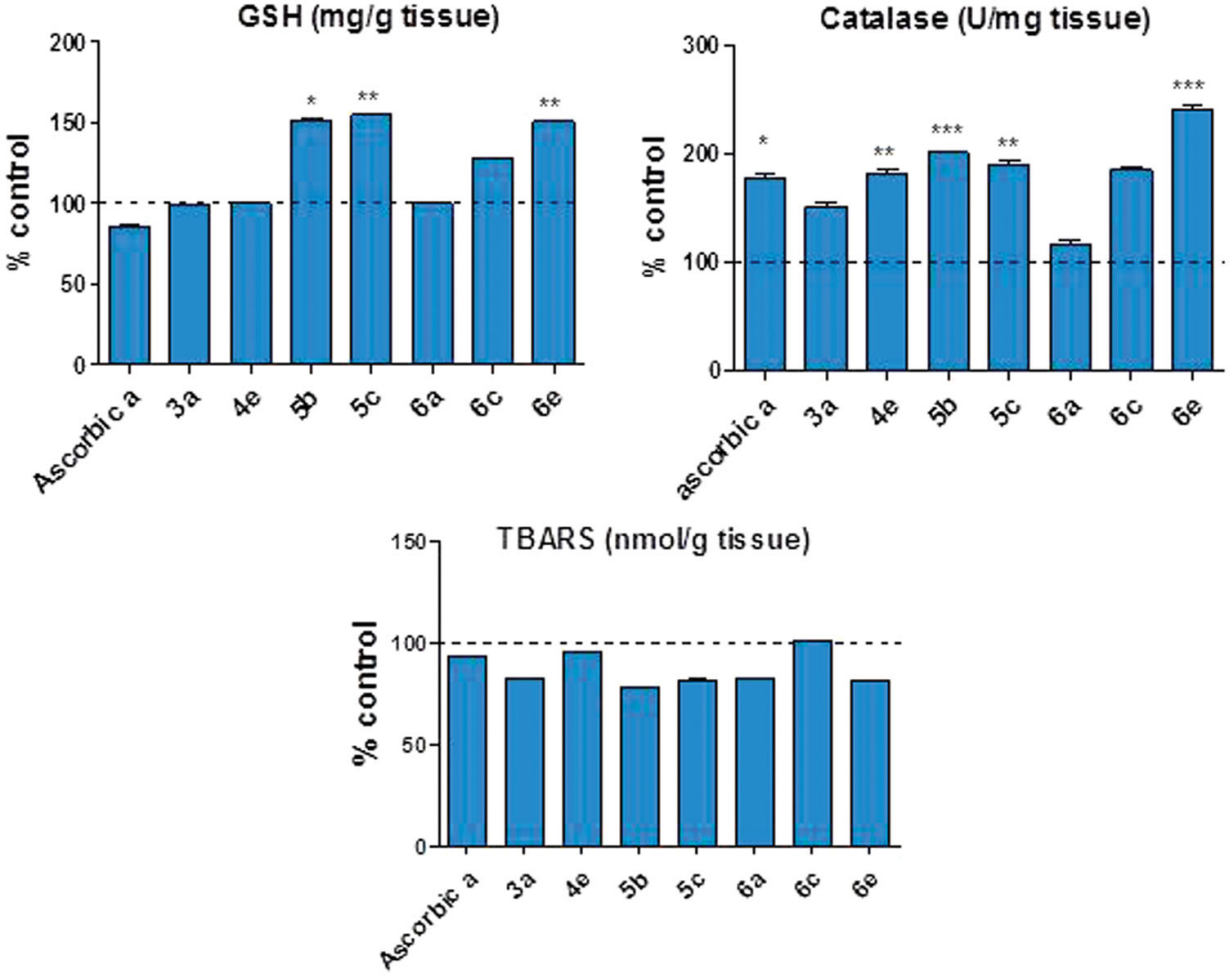
Effect of compounds **(3a, 4e, 5 b, 5c, 6a, 6c, 6e)** and ascorbic acid on the endogenous antioxidant status of rats. GSH: reduced glutathione; TBARS: thiobarbituric acid reactive substances. Data are expressed as mean ± SEM% control. (*n* = 6). *, **, and *** *p* < 0.05, *p* < 0.01, and *p* < 0.001 compared to control group.

Measuring the *in vitro* antioxidant ability of the synthetic compounds was not enough to estimate their antioxidant effects in biological systems. *In vivo* antioxidant assays could reflect the related biological implications of dietary consumption, such as effects on antioxidant enzymes and oxidation-related metabolic pathways. It is well known that lipid peroxidation is a complex process which occurs as a result of the interaction between molecular oxygen and polyunsaturated fatty acids. These free radicals can cause the oxidation of biomolecules (e.g. protein, lipid, and DNA) leading to cell injury and death^70^. Lipid peroxidation in biological systems can lead to various pathological consequences^71^. The end products of lipid peroxidation are reactive aldehydes, such as MDA, which are highly toxic to cells^72^. In addition, the MDA can react with biomolecules and exert cytotoxic, genotoxic, and neurodegenerative disorders. Since, MDA is one of the end products of lipid peroxidation, thus the level of MDA can indicate the degree of lipid peroxidation in the body. In Fact, GSH provides the first line of body defence by scavenging ROS or by acting as a co substrate in the GPx-catalysed reduction of H_2_O_2_ and lipid peroxides. Oxidative stress readily oxidises GSH to glutathione disulphide by free radicals and ROS causing depletion of GSH level^73^. Moreover, the endogenous antioxidant enzymes such as SOD removes the superoxide anion^74^, while CAT catalyses the reduction of H_2_O_2_ and protects the tissues from highly reactive stabilization that may be produced from H_2_O_2_. Pyrazole derivatives (**3a, 4e, 5b, 5c, 6a, 6c, 6e**) that showed promising *in vitro* antioxidant activities were subjected to *in vivo* study. In the acute toxicity study, the orally administered compounds did not show toxic effects in doses up to 100 mg/kg B.W. Oral administration of test compounds for 3 days increased CAT activity and GSH level and decreased the MDA concentration in the liver, which indicated that they could enhance the antioxidant status as presented in Table 2 and Figure 6. This validates the potent *in vitro* antioxidant activity shown by these compounds. However, only compounds (**5b, 5c,** and **6e**) showed significant potent antioxidant activity compared to control group at dose of 100 mg/kg. This may be attributed to the short treatment period of the animals. The results of our study showed that treatment of animals by those compounds significantly increased the level of CAT enzyme by about 101, 89, and 141%, respectively, compared to control group. The increased level of CAT leads to break down of H_2_O_2_ and prevent further generation of free radicals. The increase in intracellular thiol-based antioxidant GSH was by about 52, 55, 51%, shown by compounds (**5b, 5c**, and **6e**). The antioxidant activity may be due to potent radical-scavenging activity of isoxazoline and carbothioamide pyrazoline.

**Table 2.** *In vivo* antioxidant potential of compounds.

Compounds	CAT (U/mg tissue)	GSH (mg/g tissue)	TBARS (nmol/g tissue)
Control	23.99 ± 3.83	7.07 ± 0.50	0.422 ± 0.04
Ascorbic a	42.56 ± 3.76*	8.25 ± 0.45	0.392 ± 0.07
**3a**	36.21 ± 3.49	7.00 ± 0.27	0.350 ± 0.05
**4e**	43.48 ± 4.51**	7.05 ± 0.56	0.402 ± 0.04
**5b**	48.19 ± 1.24***	10.71 ± 0.86*	0.330 ± 0.02
**5c**	45.42 ± 4.30**	10.92 ± 0.96**	0.346 ± 0.01
**6a**	27.97 ± 3.46	7.07 ± 0.35	0.348 ± 0.03
**6c**	44.38 ± 2.81	9.01 ± 0.72	0.428 ± 0.02
**6e**	57.84 ± 3.50***	10.65 ± 0.97**	0.344 ± 0.05

CAT: catalase; GSH: reduced glutathione; TBARS: thiobarbituric acid reactive substances values are mean ± SEM. *, ** and *** *p* < 0.05, *p* < 0.01, and *p* < 0.001 as compared to control (*n* = 6).

### 3.5. Molecular modelling

**Figure 7.**
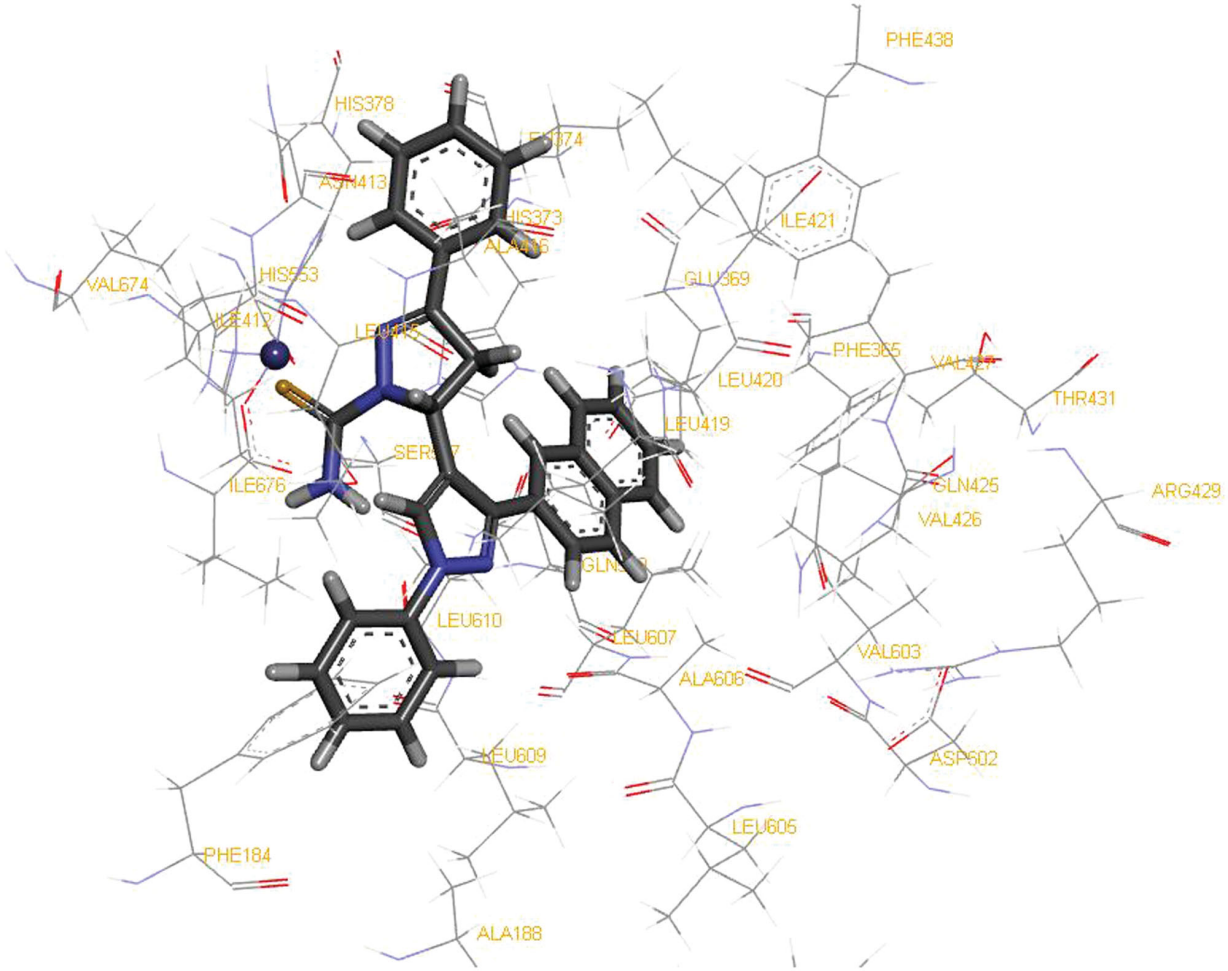
Molecular modelling of 15-LOX inhibitors **6a** (coloured by element), into the active binding site of human 15-LOX (PDB: 4NRE), tagging the protein residues that coordinate with Fe^3+^ catalytic metal (blue ball) and that interacted with the inhibitors.

To investigate the orientation of the most potent compound **6a** into the active binding site pocket of the human 15-LOX (PDB: 4NRE)^75^ and to view the inhibitor-receptor interactions, molecular modelling study was performed. All the modelling experiments described here were performed by using the DS version 4.5 (Accelrys Inc., San Diego, CA, USA). The required pdb coordinates were downloaded from the Brookhaven website (www.rcsb.org). The hydrogen atoms were then added to both the small molecule and the protein. The atom and bond types as well as the protonation state for the small molecule and the binding site were checked and corrected when needed. Water molecules were deleted. This was followed by minimizing the complex with the DS force field by using the default parameters. Analysis of the proposed binding of inhibitor **6a** (Figure 7) into the catalytic binding site revealed that both naphthalene ring and the close by phenyl ring on the pyrazole group was directed towards the hydrophobic cavity of the active binding pocket making hydrophobic-hydrophobic interactions. In addition, on the other pyrazole group, the S-atom was found to be directed towards the catalytic Fe^3+^ of the active site and the phenyl ring is making π–cationic interactions with the catalytic Fe^3+^ of the active site as well as π–π interactions with the His378 amino acid residue. It also was observed that the terminal NH_2_ group is able is make a weak H-bond (3.7 Å) with the carbonyl of the Ile 676 amino acid residue.

## 4. Conclusions

In summary, novel hybrids containing pyrazole, naphthalene and pyrazoline/isoxazoline pharmacophore were synthesized and investigated for their *in vitro* antioxidant activity using DPPH, NO and Superoxide radical scavenging assays as well as 15-LOX inhibition activity. One important pathway for antioxidant agents is through inhibiting lipid peroxidation that is mainly catalyzed by 15-LOX. The activity of the compound **6a** was assed towards both antioxidant and anti-LOX activities. It was found that compound **6a** showed NO scavenging activity higher than that of ascorbic acid. In addition, it showed potent anti-LOX activity by 2.2 folds compared to that of quercetin. Furthermore, compounds **5a** and **5c** showed comparable potency to ascorbic acid. Moreover, compound **4e** displayed good antioxidant activity. SAR studies showed that pyrazoline carbothioamide **6c** and pyrazolyl pyrazoline **3a** exhibited higher NO scavenging activity than pyrazolyl isoxazoline **5a** and *N*-phenyl pyrazolyl pyrazoline **4e**. In addition, compounds **2c, 3a, 3e, 4a, 4c, 5e, 6a, 6b, 6c, 6d,** and **6e** showed potential 15-LOX inhibition activity which was almost aligned with DPPH assay results only but was conflicted for some compounds such as **2c, 3e, 4a, 4c, 5e, 6b,** and **6d** with the other antioxidant assays. Interestingly, carbothioamides **6a** and **6e** were the most potent compounds with 2.2 and 2.1 folds that of quercetin, respectively, and almost showed similar potential antioxidant activity in all three assays. Furthermore, compounds **3a, 4e, 5b, 5c, 6a, 6c,** and **6e** showed significant RSA in all three *in vitro* assays in comparison with ascorbic acid along with 15-LOX inhibition potency using quercetin as standard suggesting an important influence of EDGs (CH_3_, OCH_3_) and di-halogen (di-Cl) on the benzene ring. Regarding heterocyclic pharmacophore, pyrazoline carbothioamide and pyrazoline showed higher RSA and 15-LOX inhibition potency than *N*-phenyl pyrazoline and isoxazoline and these observations should be taken in consideration for future designed LOX inhibitors. Furthermore, the *in vivo* results supported the *in vitro* data, i.e. compounds **5b, 5c** and **6e** at dose of 100 mg/kg B.W showed significant *in vivo* antioxidant activity through increased CAT activity, GSH level and decreased lipid peroxidation in the treated rat liver compared to control treatment. This indicates their role in enhancing the antioxidant status. These data validate the potent *in vitro* antioxidant activity shown by those derivatives. Docking study of the most potent candidate **6a** revealed that stabilization of the ligand inhibitor through hydrophobic-hydrophobic interactions. In addition, π–cationic interactions with the catalytic Fe^3+^ of the active site as well as π–π interactions with the His378 amino acid residue might be required for the potential activity of 15-LOX inhibitor. In conclusion, the obtained results suggest that these potent compounds may serve as lead candidates for 15-LOX inhibitors. Furthermore, the designed pyrazole hybrid scaffold is an interesting antioxidant pharmacophore and considered as novel lead scaffold for any future optimization.
